# A Feasible Alternative Strategy Targeting Furin Disrupts SARS-CoV-2 Infection Cycle

**DOI:** 10.1128/spectrum.02364-21

**Published:** 2022-02-09

**Authors:** Tanmoy Mondal, Gururaj Shivange, Alaa Habieb, Jogender Tushir-Singh

**Affiliations:** a Department of Medical Microbiology and Immunology, University of California, Davis, California, USA; b Department of Biochemistry and Molecular Genetics, University of Virginia, Charlottesville, Virginia, USA; c UC Davis Comprehensive Cancer Center, University of California, Davis, California, USA; University of Arizona

**Keywords:** antibody, COVID-19, SARS-CoV-2, proteases

## Abstract

The COVID-19 causing coronavirus (SARS-CoV-2) remains a public health threat worldwide. SARS-CoV-2 enters human lung cells via its spike glycoprotein binding to angiotensin-converting enzyme 2 (ACE2). Notably, the cleavage of the spike by the host cell protease furin in virus-producing cells is critical for subsequent spike-driven entry into lung cells. Thus, effective targeted therapies blocking the spike cleavage and activation in viral producing cells may provide an alternate strategy to break the viral transmission cycle and to overcome disease pathology. Here we engineered and described an antibody-based targeted strategy, which directly competes with the furin mediated proteolytic activation of the spike in virus-producing cells. The described approach involves engineering competitive furin substrate residues in the IgG1 Fc-extended flexible linker domain of SARS-CoV-2 spike targeting antibodies. Considering the site of spike furin cleavage and SARS-CoV-2 egress remains uncertain, the experimental strategy pursued here revealed novel mechanistic insights into proteolytic processing of the spike protein, which suggest that processing does not occur in the constitutive secretory pathway. Furthermore, our results show blockade of furin-mediated cleavage of the spike protein for membrane fusion activation and virus host-cell entry function. These findings provide an alternate insight of targeting applicability to SARS-CoV-2 and the future coronaviridae family members, exploiting the host protease system to gain cellular entry and subsequent chain of infections.

**IMPORTANCE** Since its emergence in December 2019, COVID-19 has remained a global economic and health threat. Although RNA and DNA vector-based vaccines induced antibody response and immunological memory have proven highly effective against hospitalization and mortality, their long-term efficacy remains unknown against continuously evolving SARS-CoV-2 variants. As host cell-enriched furin-mediated cleavage of SARS-CoV-2 spike protein is critical for viral entry and chain of the infection cycle, the solution described here of an antibody Fc-conjugated furin competing peptide is significant. In a scenario where spike mutational drifts do not interfere with the Fc-conjugated antibody's epitope, the proposed furin competing strategy confers a broad-spectrum targeting design to impede the production of efficiently transmissible SARS-CoV-2 viral particles. In addition, the proposed approach is plug-and-play against other potentially deadly viruses that exploit secretory pathway independent host protease machinery to gain cellular entry and subsequent transmissions to host cells.

## INTRODUCTION

**FIG 1:**
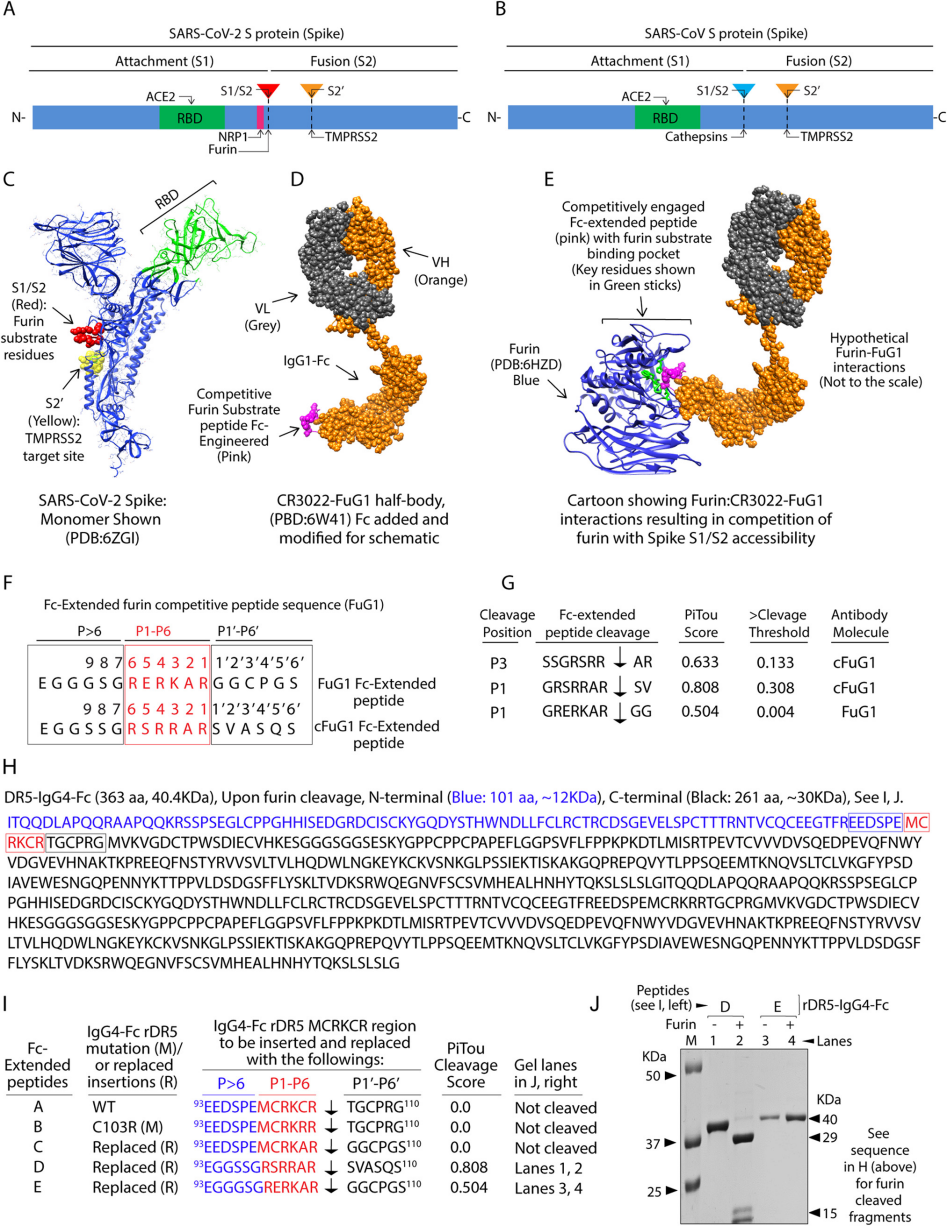
Design of furin competitive FuG1 strategy. (A, B) Schematic of SARS-CoV-2 and SARS-CoV show RBD domain, S1/S2, and S2' sites. (C) Ribbon structure of SARS-CoV-2 spike monomer (PDB: 6ZGI). Red spheres: S1/S2 represent furin substrate residues. Yellow spheres: S2' residues. RBD domain is in green. (D) 22-IgG1 (PDB: 6W41) half-body schematic inserted with Fc extendable linkers harboring competitive furin engaging residues (pink, cartoon only). (E) Schematic showing competitively engaged active site cleft of furin (PDB: 6HZD) with Fc extended peptide (pink) of 22-FuG1 antibody. VH, gold; VL, gray; furin, blue ribbon with key substrate-binding pocket residues (H194, S253, P256, N295, S368) shown as green stick model (16). (F) Sequences of FuG1 (optimal competitive lead) and cFuGI (control cleavable) Fc-extended linkers. (G) Cleavage score of FuG1 and cFuG1 Fc-extended linkers based on PiTou algorithm. (H) The amino acid sequence of human IgG4-Fc tagged recombinant DR5. The sequence in blue represents 101 amino acids (∼12KDa) at the N-terminal of DR5 MCRKCR (P1-P6) residues (red), and the sequence in black is 261 amino acids (∼30KDa), C-terminal to MCRKCR. (I) Amino acids in blue (EEDSPE), red (MCRKCR), and black (TGCPRG) of IgG4-Fc tagged DR5 were replaced with the corresponding sequences shown to generate peptides A to E. P1 to P6 residues are part of the core furin substrate site, *P* > 6 represent residues upstream of the core furin substrate site, and P1’ to P6’ represent residues downstream of the core furin substrate site. Furin cleavage score based on PiTou tool composed on hidden Markov model is shown for each 18 amino acid peptides. (J) Based on the PiTou score, various IgG4-Fc tagged DR5 with indicated substituted peptides (B to E) were expressed, purified, and dialyzed in phosphate-buffered saline (PBS), followed by cleavage analysis using 50 ng recombinant furin. Recombinant DR5 inserted with Peptide-D showed expected furin cleavage and the release of ∼30KDa and ∼12KDa fragments (lane 1 and 2; see H for size details), while rDR5 having peptide-E (lane 3 and 4) was not cleaved by recombinant furin.

As of January 1, 2022, SARS-CoV-2 has caused >290 million infections and >5.44 million deaths worldwide. Despite the remarkable speed of multiple vaccine approval by the USFDA, the detailed mechanism of SARS-CoV-2 cellular entry, chain of infectivity, and pathology remains unclear(1). Thus, effective strategies targeting and interfering with the viral chain of infection before cellular entry remain pivotal for long-term therapeutic efficacy against continuously evolving SARS-CoV-2 mutant variants (2). Similar to SARS-CoV, SARS-CoV-2 entry into target lung cells is dependent on spike receptor-binding domain (RBD) interactions with ACE2 (3). However, unlike SARS-CoV, the SARS-CoV-2 spike protein harbors an arginine-rich multibasic site (S1/S2) between attachment (S1) and fusion (S2) domains (Fig. 1A and B). The cleavage of arginine-rich basic residues by host cellular furin protease is critical for efficient host cell membrane fusion during transmission chain (4), SARS-CoV-2 cellular entry, and infection-induced cytopathic effects into human cells and tissues (5, 6). Experimentally tested host furin protease targeting chemical inhibitor drugs (7), *de novo* peptides, ACE2 traps (8), and mini proteins (9) has been shown effective in breaking chain of viral infection in cellular models. However, due to the lack of targeting specificity against spike-positive primary lung cells and infected tissues, if tested clinically, these protease inhibitory approaches are highly likely to interfere with the normal cellular processes in the body due to their random tissue distribution (10).

The spike structural studies have demonstrated interactions of solvent-exposed furin cleaved region (S1/S2) with additional surface receptors to enhance viral cellular entry (4, 11) and disease pathogenicity (5, 6). Thus, selective targeting of furin in virus-producing cells remains one alternate strategy to break the SARS-CoV-2 infection cycle. One of the key barriers for lack of selectively targeted furin-inhibitor therapies (against SARS-CoV-2 infected and spike-producing cells) is the uncertainty of cellular organelle-specific furin mediated spike cleavage during viral cellular egress of virus (7). In light of recent findings (12) that β-coronaviruses such as SARS-CoV-2 uses lysosomal deacidification and potential late-recycling endosomal trafficking route for membrane egress rather than a trans-Golgi-network (TGN) secretory pathway, selective perturbation of furin function in spike escape route holds the key. Thus, targeting strategies capable of operating in cell membrane-endosome-lysosome networks are a rational start. As early, late, and recycling endosomes enriched immunoglobulin-G fragment crystallizable (Fc) receptor called neonatal receptor (FcRn) is vital in regulating the circulating level of antibodies (13, 14), IgG1-Fc mediated targeting of furin remains untested in the context of spike S1/S2 processing. The present study demonstrates biosynthetic secretory pathway independent S1/S2 cleavage of spike protein by furin. Importantly, to selectively target furin-driven spike processing of SARS-CoV-2, we engineered and described a simple, compelling, and targeted IgG1-Fc-based approach that interferes with the regulatory furin protease function to inhibit fusion-ready S2 fragment generation and to destabilize full-length spike (S0) protein.

## RESULTS

### The FuG1 strategy.

We hypothesize that a strategy capable of competitively inhibiting cellular furin and selectively targeting SARS-CoV-2 in the infected viral-producing cells is critical for therapeutic specificity. Thus, we engineered a spike targeting IgG1-Fc-based design that would selectively compete with early, late, recycling endosomal, and cell surface enriched furin protease function at the site of potential spike trafficking, processing, and incorporation into the virus. The latter would significantly interfere with S1/S2 cleavage of the full-length spike (S0) to generate viral fusion ready S2 (S0: ∼180KDa to S2: ∼80KDa) fragment (3, 15). As a result, regulated spike viral incorporation and function in virus-producing cells would be significantly impeded to negatively impact the subsequent host-cell entry and SARS-CoV-2 chain of infection function. The simultaneous spike-targeting antibody Fab and Fc-extended peptide capable of competitively inhibiting furin substrate-binding pocket (16) are shown as a schematic illustration in Fig. 1C to E (see figure legends for details). We name this targeting approach “FuG1,” an anti-spike IgG1 that competitively inhibits furin via engineered and flexibly linked Fc-extended peptide beyond the CH3 domain.

### Selection, design, and flexibility of optimal furin inhibiting FuG1 peptide.

To design optimally competitive and furin engaging Fc-extended peptide in FuG1, we used the previously described PiTou tool composed of a hidden Markov model (17). The PiTou score ranges from 0.01 to 0.99 (17); however, the scores in the higher range (>0.7) accurately predict furin cleavage with the highest efficiency (17). Specifically, we enriched the P1′ to P6′ region of the 18 amino acid Fc-extended peptide in FuG1 with nonpolar hydrophobic residues (Fig. 1F), including a side-chained cysteine (GGCPGS), as enrichment of hydrophobic residues (over hydrophilic) near the P3′ to P6′ region has been described to interfere with furin target cleavage efficiency (17). Besides, acidic anchor glutamate (E) was inserted at P5/P6 position, as the latter has shown to significantly inhibit furin catalysis without affecting the binding (18). As previous studies (17, 19) have suggested minimal loss of furin binding against computationally predicted target sequences having PiTou score close to cleavage threshold (0.5), we chose 18 amino acid Fc-extended peptide with a score of 0.504 to inhibit furin competitively (Fig. 1F and G). Next, based on a range of PiTou scores, various Fc-extended peptides were inserted into recombinant death receptor-5, rDR5-IgG4-Fc (20) by replacing the region near its patch of positively charged residue motif (RKCR) (21) in the third cysteine-rich domain (Fig. 1H and I, see the sequences in blue, red, and black). These constructs were expressed using CHO cells in the presence of furin inhibitor. If rDR5-IgG4-Fc were to be cleaved by recombinant furin, two approximately ∼30KDa and ∼12KDa fragments would be generated (Fig. 1H). The selected lead FuG1 peptide (Peptide-E, PiTou score of 0.504), when inserted in rDR5-IgG4-Fc, was not cleaved by recombinant furin *in vitro* (Fig. 1J, lane 3 and 4). The furin optimal Fc-extended peptide (Peptide-D, PiTou score of 0.808) engineered with the sequence of spike S1/S2 residues (SVASQS) in the P1′ to P6′ region, when inserted in rDR5-IgG4-Fc resulted in expected ∼30KDa and ∼12KDa fragments when incubated with recombinant furin (Fig. 1J, lane 1 and 2) (22). We chose the cleavable sequence with a 0.808 score as a control (cFuG1) for furin binding studies.

**FIG 2:**
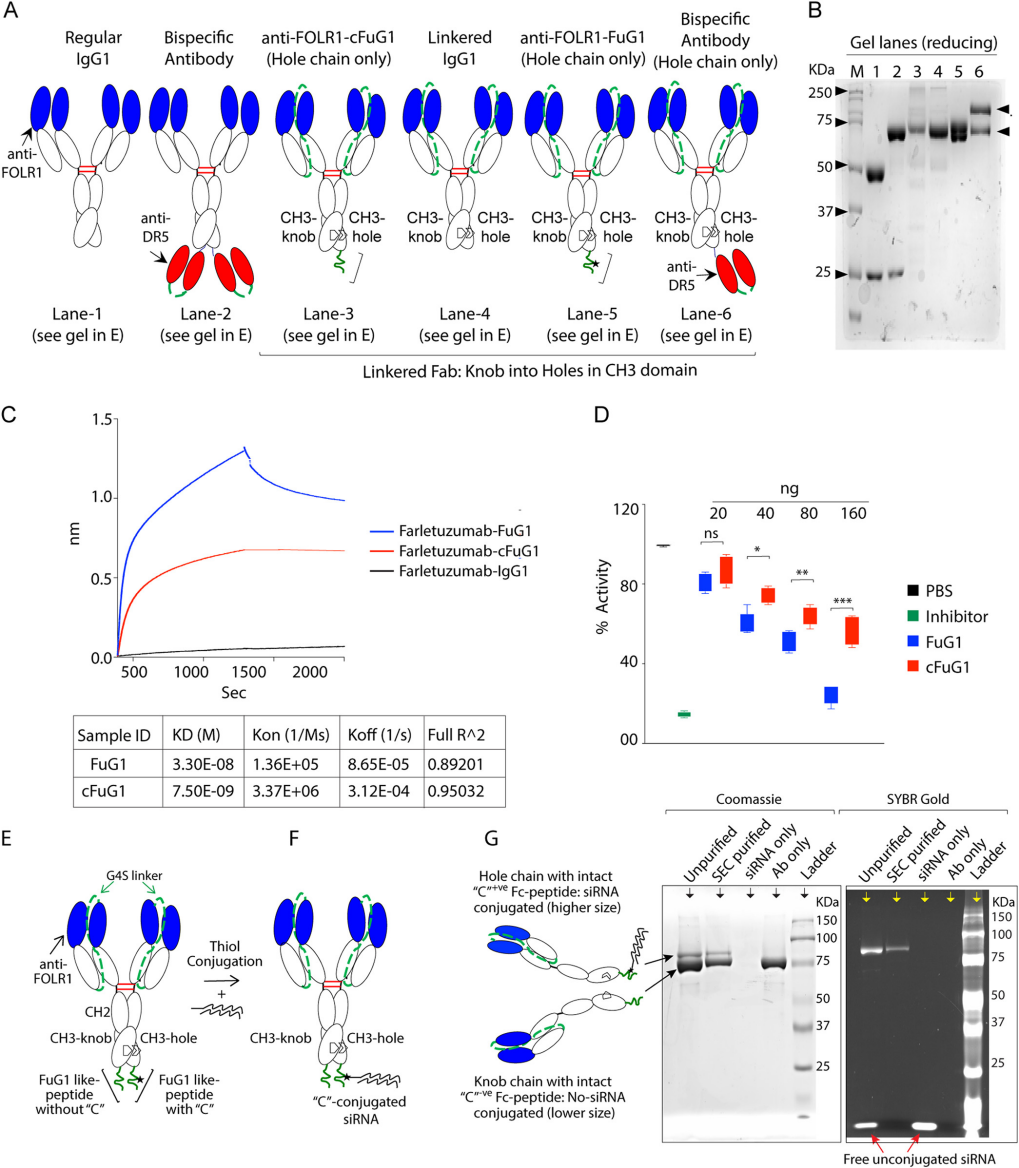
Confirmation of *in vivo* stability, furin inhibitory function, and flexibility of Fc-extended FuG1 peptide strategy. (A, B) Genetic construction schematic of various recombinant antibodies in (A) were run under reducing conditions in (B). Anti-FOLR1 antibody, farletuzumab is shown as a regular IgG1 (lane 1 in B). A 50 kDa and 25 kDa bands corresponding to heavy and light chains are evident in reduced sample in lane 1. Lane 2 is a bispecific antibody where a single chain variable fragment (scFv) was covalently generated in continuous of CH3 domain of VH, hence 75 kDa and 25 kDa bands corresponding to heavy and light chains are evident in reduced sample. Lane 3 is a linkered farletuzumab IgG1, where 3′ end of light chain is covalently linked by G4S linkers to the 5′ end of heavy chain. As a result, a single band around ∼75 kDa is evident. Lane 4 is exactly the same as lane 3 except a G4S linker and 18 amino acid cFuG1 peptide is engineered after the CH3 domain only in one chain (hole). Because the cFuG1 (Fig. 1F and G) peptide is an optimal PiTou furin substrate, it was cleaved in CHO cells during expression. As a result, size of lane 4 is similar to linkered IgG in lane 3. Lane 5 is similar to lane 4, except it contains non-cleavable FuG1 peptide with the non-optimal PiTou score (Fig. 1F and G). As a result, during CHO cell expression, two distinct bands differing only in few kDa sizes are evident. Lane 6 is same as lane 4 and 5 except instead of Fc-extended furin engaging peptides, the hole chains contains scFv similar to lane 2. Hence, the sizes in reducing gels are ∼75KDa and ∼100KDa. No VL is evident in lanes 3 to 6 in B. (C) The binding kinetics of immobilized biotinylated furin against indicated farletuzumab-FuG1, farletuzumab-cFuG1, and Farletuzumab-IgG1 were measured using BLI. (D) FuG1 and cFuG1 antibodies were tested using a competitive inhibitory furin assay kit, which measures fluorescent signal as an indicator of the protease activity kinetics. PBS and assay kit supplied inhibitor (chloromethylketone) plots show optimal and competitively inhibited furin activity range with the supplied substrate. Farletuzumab-cFuG1 and farletuzumab FuG1 with indicated concentrations were added to competitively inhibit furin activity in the presence of supplied substrate, followed by readout of plates for fluorescent signal using microplate reader at 380 nm/460 nm (see also Materials and Methods section). (E to G) Farletuzumab with a Fc-conjugated peptide similar to FuG1 with asymmetric cysteine only in Fc-extended hole chain was conjugated with siRNA under the non-reducing conditions as per manufacture protocol. Following the thiol conjugation reaction (F), the conjugates were purified using size exclusion chromatography. Both purified and crude conjugates were separated on reducing gel and control siRNA alone and antibody alone (G). Lane 1 and lane 2 showed two bands confirming the increase in size due to conjugated siRNA. The same gel was confirmed by SYBR-gold staining to confirm the higher size band representing conjugated with siRNA. Unconjugated siRNA was only present in unpurified crude samples at the bottom. Lane 3 and 4 have free unconjugated siRNA and antibody controls loaded for size.

As furin is ubiquitously expressed in endosomal, secretory, and membrane compartments of CHO cells, no cleavage of lead FuG1 peptide after genetically linking to the CH3 domain of an IgG1-Fc by G4S linker during the protein expression conditions, will support its *in vivo* stability and structural integrity. In similar conditions, cleavage of cFuG1 peptide (Fig. 1I and J) will indicate *in vivo* instability of the latter. To this end, we used an anti-folate receptor alpha-1 (FOLR1) targeting a clinical antibody named farletuzumab (20). We engineered farletuzumab with linkered Fab domains to harbor either Fc-extended lead FuG1 or control (cFuG1) like peptide sequences in only one heavy chain with CH3-hole mutations as described earlier by our group and others (20, 23) (Fig. 2A). These, along with multiple control antibodies (Fig. 2A), were expressed using CHO suspension cultures without furin inhibitor (20). When tested in reducing SDS-PAGE gel, the lead FuG1 like peptide containing linkered farletuzumab showed two clear expected size bands, the larger with genetically linked peptide, while smaller without genetically linked flexible peptide (compare lane 4 vs 5). As per PiTou prediction (Fig. 1I and J) and our expected *in vitro* cleavage data (Fig. 1J), cFuG1 like peptide containing linkered farletuzumab showed only one band (Fig. 2A and B, lane 3, 4 vs 5 of gel), as these linkered Fabs have VL also genetically linked to VH as described earlier (20). A 25KDa VL band was evident in non linkered farletuzumab monospecific and bispecific antibodies (Fig. 2A and B, lane 1, 2). These results support *in vivo* stability of genetically linked FuG1 peptide to the CH3 domain of an IgG1-Fc.

To confirm furin binding and competing function, farletuzumab-FuG1 and farletuzumab-cFuG1 were expressed in the presence of furin inhibitors. Purified antibodies were analyzed for their Fc-extended peptide binding to biotinylated furin using BLI. Although furin binding affinity (K_D_) and association rate (K_ON_) of FuG1 were lower (compared with cFuG1), the furin dissociation rate (K_OFF_) of FuG1 was also almost 10-fold reduced (Fig. 2C). The latter indicated a potential higher competitive furin occupancy time of FuG1 peptide with the increased antibody concentration (24). In support, when tested using an *in vitro* protease assay that relies on the generation of the fluorogenic substrate upon cleavage by recombinant furin (25), we observed significant inhibition by Farletuzumab-FuG1 antibody, particularly at higher doses when compared with the control farletuzumab-cFuG1 containing peptide (Fig. 2D). As the Fc-extended FuG1 peptide contains cysteine (C) in the P1′ to P6′ region (Fig. 1F), if expressed asymmetrically only in one heavy chain, the cysteine (C) should be available for conjugation in non-reducing conditions (using thiol chemistry). To this end, we expressed farletuzumab with asymmetric cysteine only in the Fc-extended peptide (similar to the FuG1 peptide described in Fig. 1F) of the hole VH chain (Fig. 2E). Purified antibody was subjected to covalent conjugation with reactive thiol CY5 labeled locked siRNA (LNA) in non-reducing conditions (Fig. 2F) as described in detail earlier (26). Given that thiol conjugation of locked siRNA (LNA) has been shown to increase the VH size by ∼15KDa (26), as expected, the siRNA conjugated farletuzumab hole chain had a larger size (Fig. 2G). The siRNA conjugation was further confirmed by making use of SYBR gold staining of the same gel. Only CY5 labeled siRNA in the corresponding larger size lane showed the SYBR gold signal (Fig. 2G, right gel). Free siRNA at the bottom in lane 1 and 3 confirmed RNA (but not protein) specificity of SYBR gold signal. Collectively the results so far support *in vivo* stability (Fig. 2B), furin binding plus inhibitory function (Fig. 2C and D), and flexible availability of Fc-extended FuG1 peptide strategy (Fig. 2E to G).

### Ectopically expressed spike is furin processed independent of trans-Golgi-network.

**FIG 3:**
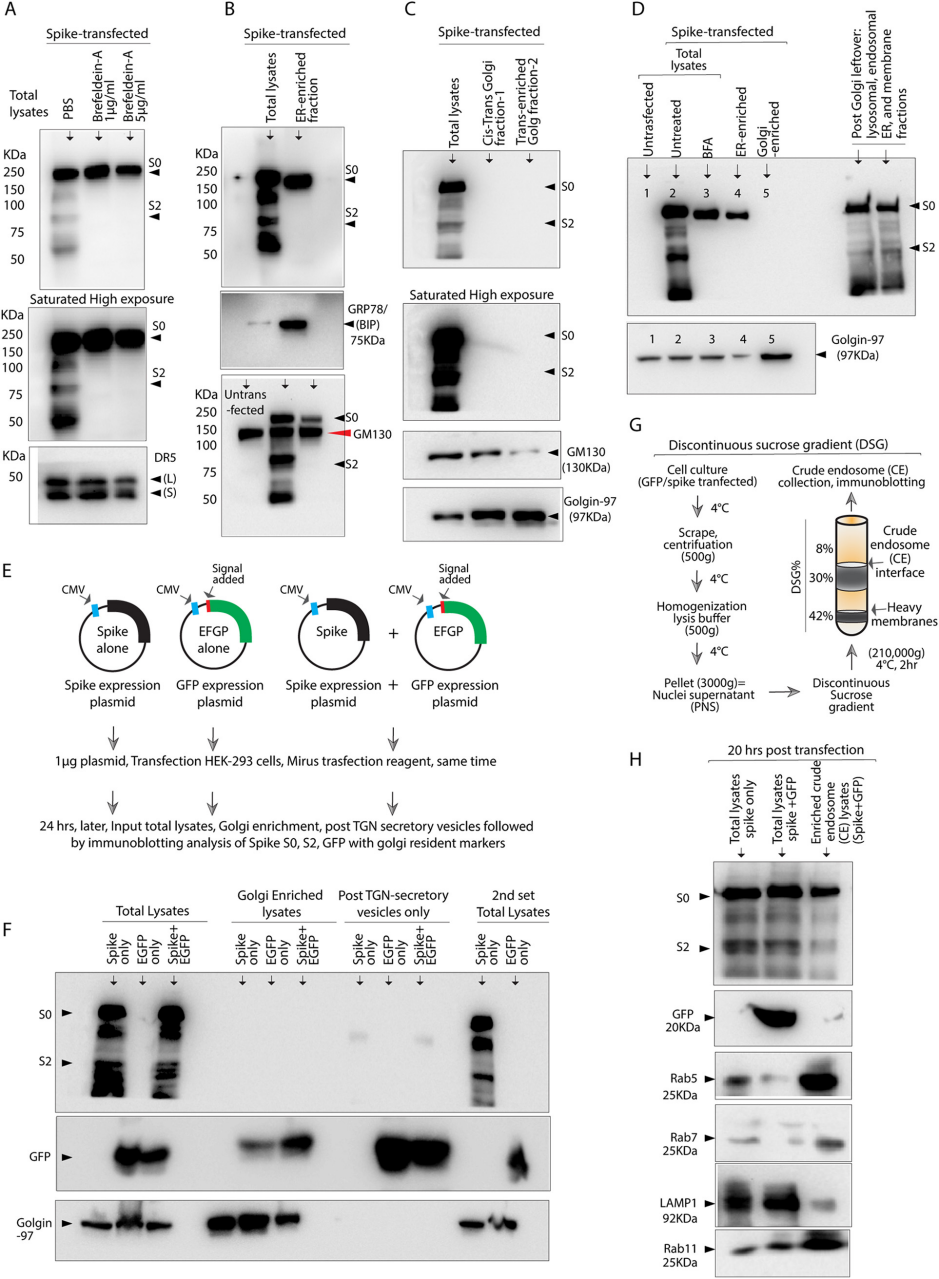
TGN independent cleavage of ectopically expressed spike. (A) 293-ACE2 cells were treated with indicated Brefeldin-A concentrations at the time of spike transfection. Lysates were analyzed for spike and DR5 24 h later using immunoblotting. (B to D) Cells grown in 150 cm^2^ dishes (divided in groups as indicated on top) were transfected with the spike. ER and Golgi fractions were enriched 24 h later using Minute ER and Golgi enrichment kits (see Materials and Methods section) as indicated on top. Lysates were subjected to immunoblotting with indicated ER and Golgi resident markers along with spike. (E, F) Same as B-D, except along with spike, GFP (with N-terminal membrane sorting peptide) was used as a positive control for traditional TGN sorting. (G) Schematic of crude endosome (CE) enrichment assay for data shown in (H) (see Materials and Methods section). (H) Along with spike and spike+GFP total lysates, enriched CE from spike+GFP co-transfected cell were analyzed for indicated endosomal, lysosomal marker using immunoblotting.

It is believed that trans-Golgi-network (TGN) enriched furin cleaves spike at S1/S2 sites. However, no study has conclusively proved the selective role of the TGN secretory pathway in spike processing, despite the well-established additional regulatory furin protease functions in early, late, recycling endosomes and plasma membrane in mammalian cells (27, 28). Specifically, adaptor proteins and regulatory modifications of protein tethers regulate TGN independent furin activity in sorted late or recycling endosomes and at plasma membrane independent of TGN function (28–30). Thus, various furin processing mechanisms exist to generate functional effector proteins (29). In light of recently published studies of TGN independent lysosomal deacidification route of SARS-CoV-2 (12), we sought to investigate if spike S1/S2 cleavage event happens in TGN or post-TGN endosomal-cell surface recycling route. To this end, spike transfected HEK-293-ACE2^+^ and HEK-293-ACE2- cells were treated with endoplasmic reticulin (ER) to TGN trafficking inhibitor Brefeldin-A (Bfa). Bfa completely inhibited spike S1/S2 cleavage (Fig. 3A), ruling out ER cleavage of the spike, which was reconfirmed via enriching ER-resident chaperone GRP78/BIP containing fractions (31) (Fig. 3B). Next, we enriched both cis and trans-Golgi compartments to analyze S1/S2 cleavage using the cell fractionation kit. Surprisingly, despite repeated testing, full-length spike (S0) expression was not detected in TGN compartments even though a significant accumulation of cis and trans-Golgi compartments resident markers GM130 and golgin-97, respectively, was consistently evident (Fig. 3C and D). To further reconfirm these studies, we co-transfected spike expression plasmid and GFP expression plasmid (GFP with N-terminal membrane signal peptide) together and separately followed by Golgi compartments and post TGN secretory vesicle enrichment (Fig. 3E). When these fractions were immunoblotted for spike and GFP, GFP protein followed the traditional route of shuttling from TGN to secretory vesicle independent of being expressed alone or together with the spike protein (Fig. 3F). On the contrary, despite enrichment of GFP and resident golgin-97 protein, spike signal was completely absent in TGN and post TGN secretory vesicles (Fig. 3F). These results strongly support TGN-independent sorting of spike (12) and underpin lysosomal deacidification followed by late, early and recycled endosomal exit route to plasma membrane. Besides, spike cleavage was evident when after TGN fractionation, leftover intracellular membrane fractions (other than Golgi and plasma membrane) that include lysosomes, endosomes, mitochondria, and ER were enriched (Fig. 3D, last two lanes). To further demonstrate lysosomal/endosomal system-mediated spike cleavage, we enriched crude endosomal (CE) fractions via discontinuous sucrose gradient as described earlier (32) (Fig. 3G, see Materials and Methods). Evidently, early (Rab5), recycling (Rab11), and late endosomal (Rab7) resident proteins enriched in CE fraction, with visible spike cleavage (Fig. 3H).

### FuG1 peptide does not interfere with the target binding and cellular function of antibodies.

**FIG 4:**
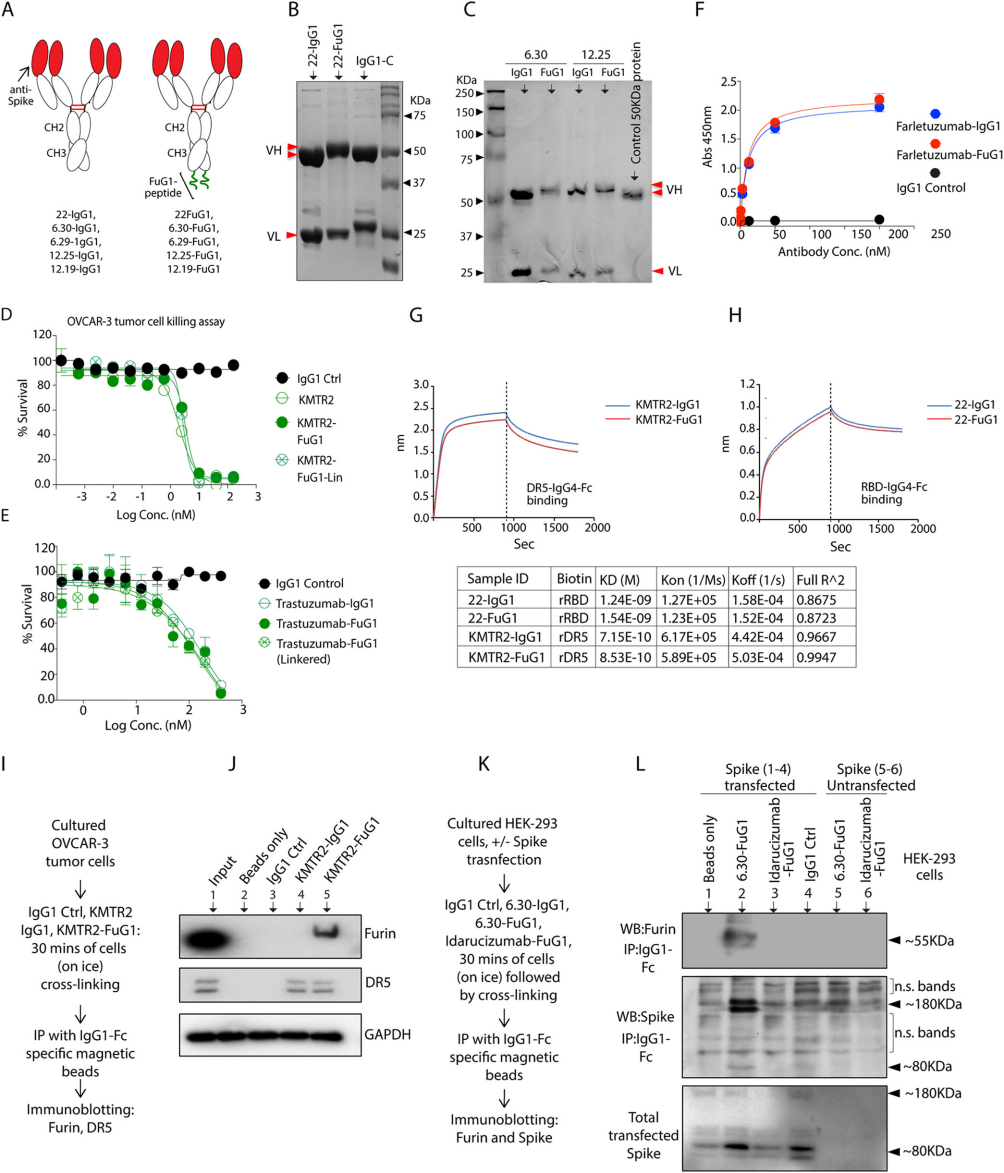
Fc-extended peptide does not interfere with target binding and activity FuG1 antibodies. (A) Genetic construction schematic of 22-IgG1, 22-FuG1. Fc extended FuG1 peptide is shown in green. (B) A reducing SDS-Gel of 22-IgG1 (lane 1) and 22-FuG1 (lane 2). Red arrows depict the difference in the sizes of IgG1 and FuG1 VH. (C) A reducing SDS-Gel of 6.30 and 12.25 IgG1 and FuG1 antibodies. Red arrows show the difference in the sizes of IgG1 and FuG1 VH. (D) Cell viability assay of KMTR2-IgG1, KMTR2-FuG1, and KMTR2-FuG1 with linkered Fab against ovarian cancer cells. (E) Cell viability assay of trastuzumab-IgG1, trastuzumab-FuG1 and trastuzumab-FuG1 with linkered Fab against breast cancer cells. (F) IgG4-Fc tagged rFOLR1 antigen was coated on 96-well plates overnight. Coated plates were treated with the increasing concentrations of either farletuzumab-IgG1 or farletuzumab-FuG1 and IgG1 control antibodies as indicated. Following numerous washes, the HRP conjugated secondary antibody that is specific to IgG1-Fc (but not IgG4-Fc) was used to measure the binding strength using TMB substrate and ELISA plate reader capable of reading at 450 nm (*n* = 3). (G, H) The binding kinetics of immobilized biotinylated recombinant DR5-IgG4-Fc and RBD IgG4-Fc against indicated antibodies were measured using BLI. (I) Schematic of the experiment shown in (J). (J) DR5 expressing ovarian (OVCAR-3) tumor cells were treated with indicated antibodies for 30 min in cross-linking conditions. IP was carried out using IgG1-Fc specific magnetic beads followed by immunoblotting against DR5, furin. (K) Schematic of the experiment shown in (L). (L) Spike transfected and untransfected (+/-) HEK-293 cells were treated with indicated antibodies followed by IP using IgG1-Fc specific magnetic beads. The lysates were immunoblotted with spike and furin.

**FIG 5:**
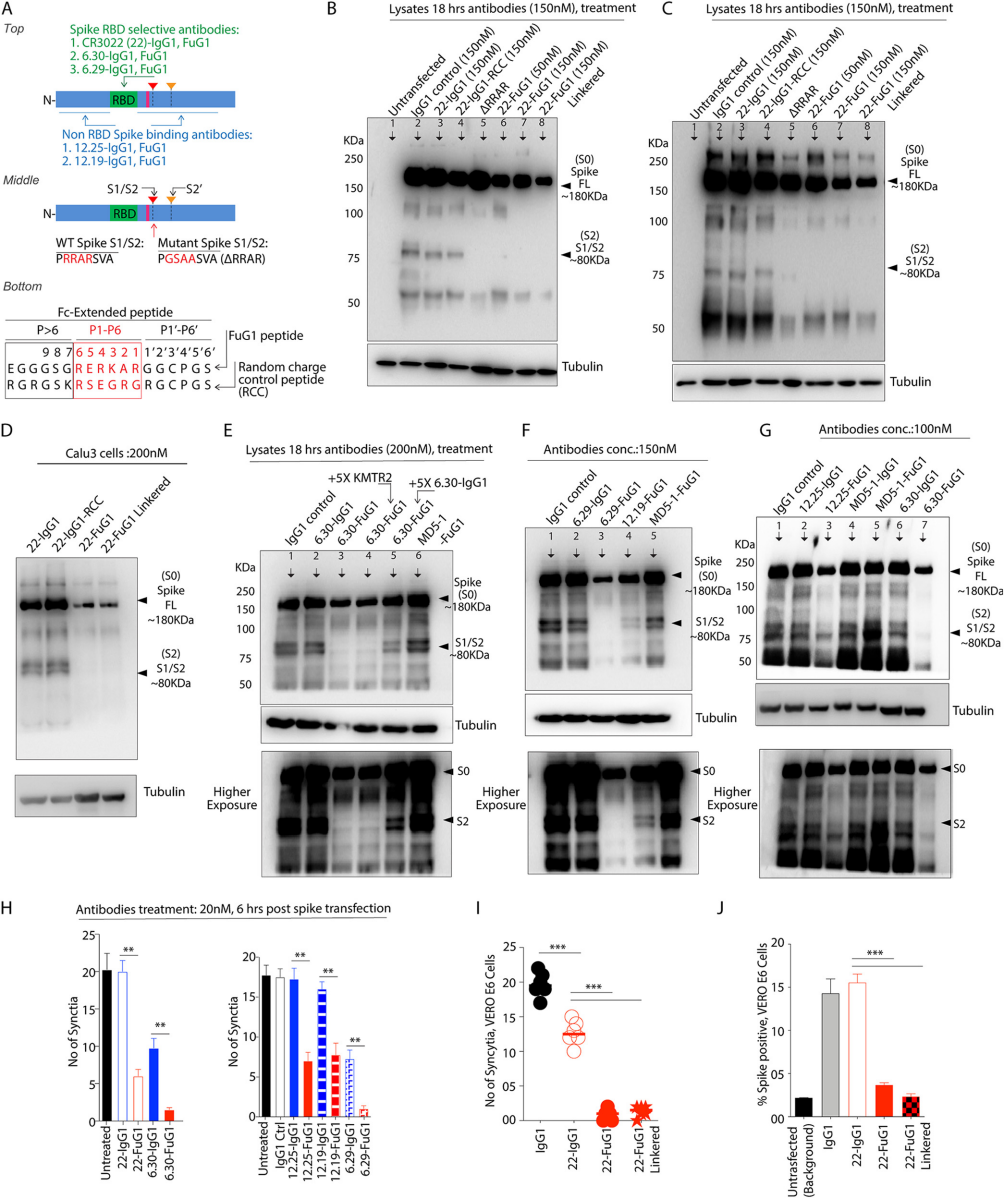
FuG1 strategy interferes with S1/S2 cleavage in spike transfected cells. (A) Top, Schematic of SARS-CoV-2 showing RBD (green) and non-RBD binding antibodies (blue). Middle, Schematic of SARS-CoV-2 showing wild type S1/S2 sequence and RRAR mutant sequence. Bottom, Sequence comparison of Fc-extended FuG1 peptide with Fc-extended random charge control (RCC) peptide. (B) 4 h post-WT spike DNA transfection, indicated antibodies were added onto the HEK-293 cells (70% to 80% confluent) and lysates were prepared after 24 h posttransfection. For control Furin mutant ΔRRAR spike plasmid was used. (C) Same as B, except ACE2-stable HEK-293 cells were used. (D) Same as C except Calu3 cells were tested. (E) Same as C except 6.30-IgG1 and 6.30-FuG1 antibodies were used. In lane 4, 5-fold KMTR2 was added along with 6.30-FuG1 and in lane 5, 5-fold 6.30-IgG1 was added to significantly out-compete with 6.30-FuG1 binding with spike. (F, G) Same as (E), except ACE2-stable HEK-293 cells were used, and instead of 22-FuG1, other spike RBD targeting (6.29-IgG1, 6.29-FuG1, 6.30-IgG1, 6.30-FuG1), and non-RBD targeting (12.19-FuG1, 12.25-FuG1) antibodies were used as indicated on the top. Murine DR5 targeting MD5-1 is specificity control. Tubulin is loading control and higher exposure blots are also shown. (H) Spike transfected 293-ACE2 cells were treated with indicated antibodies 6 h post-spike transfection. 24 h later number of syncytia was counted after various indicated treatments. Error bar indicated SEM (*n* = 3). (I) Growing VERO-E6 cells were transfected with 1.0 μg WT spike (expression vector). After 4 h of transfection, IgG1 control, 22-IgG1, 22-FuG1, and 22-FuG1-Lin (100 μg) were added to media. After 24 h, number of syncytia were counted (using EVOS imaging system) and plotted. Data indicated SEM (*n* = 3). (J) Same as I, except % surface expression of spike positive cells was analyzed after 48 h using flow cytometry. Data in (H to J) represent three independent experiments. For statistical analysis we used unpaired T-test with Welch’s correction (**, *P* < 0.005; ***, *P* < 0.0001) and each immunoblot is representative of two or three sets of experiments.

FcRn-directed and/or target antigen-mediated endocytosis results in the trafficking of surface receptor-bound antibodies into the recycling compartments (13, 14) and endosomal-lysosomal network (33). Thus, upon their endosomal trafficking, we hypothesized that anti-spike FuG1 antibodies would interfere with the spike S1/S2 cleavage via competitively saturating furin at the membrane and recycling cellular compartments. To functionally test the latter, along with SARS-CoV and SARS-CoV-2 RBD binding antibody CR3022-IgG1 (22-IgG1 hereafter), multiple other spike RBD and non-RBD targeting antibodies were converted into FuG1 strategy (Fig. 4A to C, 5A). In reducing conditions, the heavy chain (VH) of various engineered FuG1 antibodies had ∼3 kDa higher molecular weight while the light chain (VL) remains of the same size (Fig. 4B and C). Next, we confirmed that adding the Fc-extended furin linker peptide did not affect the function and binding of various FuG1 antibodies. For functional assays, we tested death-receptor-5 (DR5) targeting KMTR2-IgG1 versus KMTR2-FuG1 and human epidermal growth factor receptor-2 (HER2) targeting Trastuzumab-IgG1 versus Trastuzumab-FuG1. FuG1 conversion did not affect the function of anti-DR5 and anti-HER2 targeting clinical antibodies (Fig. 4D and E). Similar results were evident in binding assays with anti-DR5 and anti-FOLR1 targeting FuG1 antibodies (Fig. 4F and G) and spike RBD binding antibody 22-IgG1 and 22-FuG1 antibodies (Fig. 4H). We next texted FuG1 co-engagement of spike and furin in immunoprecipitation studies. When ovarian (OVCAR-3) cancer cells were tested using DR5 targeting KMTR2-IgG1 and KMTR2-FuG1 antibodies, both antibodies pulled down DR5; however, only KMTR2-FuG1 interacted with furin (Fig. 4I and J). Similarly, we performed IP with spike-RBD targeting antibody named 6.30-FuG1 (Fig. 4K, also see Fig. 5A and Materials and Methods for sequence details). Despite background on immunoblots, 6.30-FuG1 did pull down detectable S0 spike (and furin) selectively from spike transfected HEK-293 cells (Fig. 4L). The ∼80KDa (S1/S2 cleaved) fragment was also partially detectable only from FuG1 immunoprecipitated and spike transfected (Fig. 4L, middle blot) cellular lysates. We also made use of Pradaxa (a blood thinner small molecular inhibitor) targeting antibody idarucizumab (20) which has been exclusively used in our lab as a control molecule (20, 34). When tested, idarucizumab-FuG1 did not immunoprecipitated furin (Fig. 4L). As both OVCAR-3 cells (Fig. 4J) and HEK-293 cells (Fig. 4L) do not express ACE2 but immunoprecipitated furin along with corresponding target antigens (DR5 by KMTR2 and spike by 6.30), these results indicated ACE2 co-engagement independent interaction of FuG1 strategy with spike and furin.

### FuG1 interferes with optimal S1/S2 processing of spike in cultured cells.

**FIG 6:**
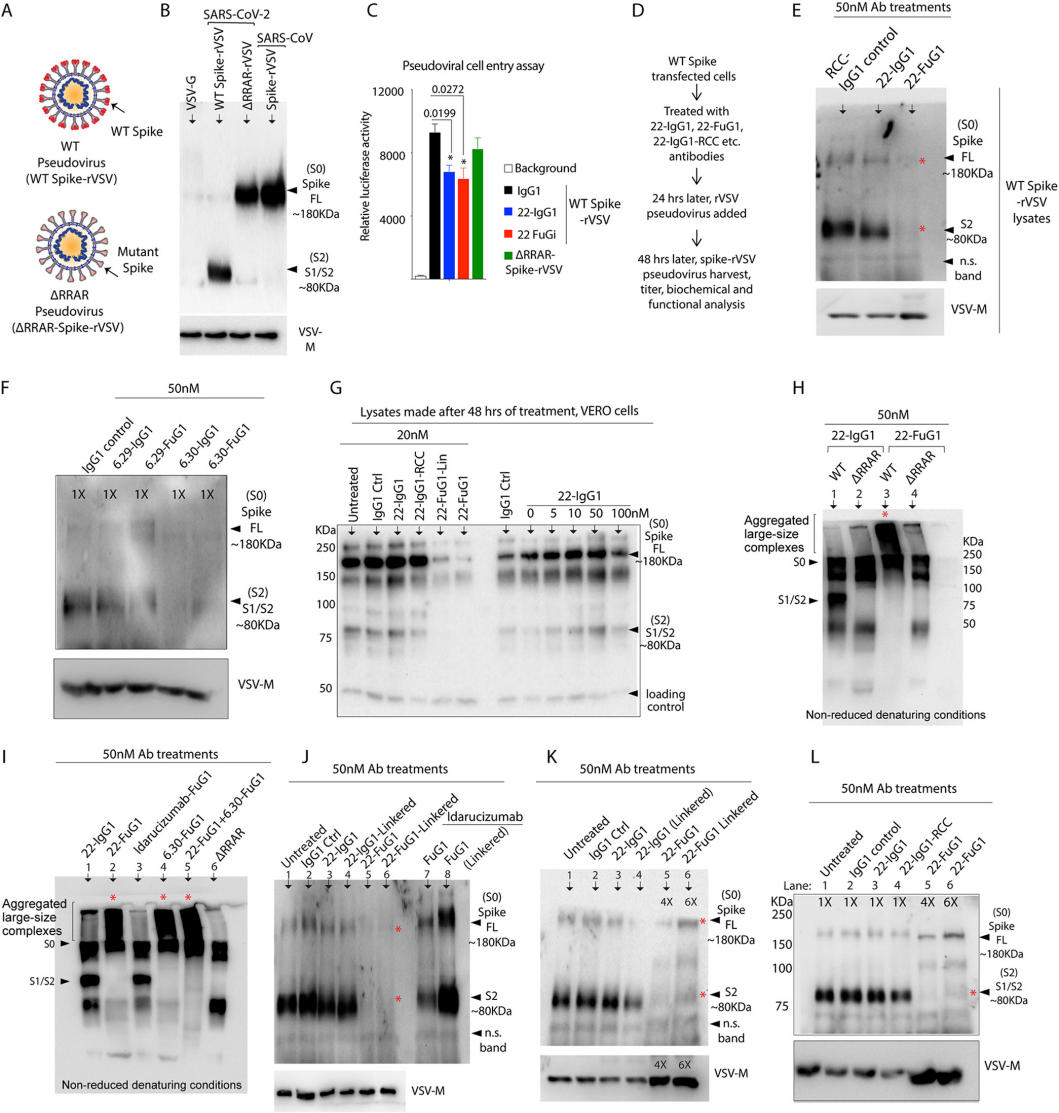
FuG1 antibodies destabilize S0 for particle incorporation in virus-producing cells. (A) Schematic of generation of WT and ΔRRAR spike expressing pseudovirus. (B) VSV-G, SARS-CoV and WT or furin mutant SARS-CoV-2 spike transfected BHK1 cells were added with replication-restricted G*ΔG-luciferase-rVSV particles. 48 h later lysates, harvested pseudovirions were analyzed for S0 and S2 fragments using immunoblotting. (C) Harvested pseudoviral particles bearing WT spike proteins were incubated with indicated antibodies for 1 h at 37, followed by transduction of 293-ACE2 cells. After 24 h, a virus-encoded luciferase signal was measured from the lysates. ΔRRAR pseudoviral particles were used as control. Error bar indicated SEM (*n* = 3). (D) Schematic workflow of experiments shown in E, F, and G. (E) Same as B, except similar to 293-ACE2 transfection experiments (Fig. 4B and C), spike transfected BHK1 cells were pretreated with indicated antibodies during pseudoviral production. (F) WT spike transfected BHK1 cells were treated with indicated antibodies (4 h posttransfection) during pseudoviral production. 48 h later lysates, harvested pseudovirions were analyzed for S0 and S2 fragments using immunoblotting. VSV-M is loading control. (G) WT spike transfected VERO-E6 cells were treated with indicated antibodies 48 h, followed by immunoblotting. (H) WT and ΔRRAR spike transfected 293-ACE2 cells were treated with indicated antibodies (4 h posttransfection). Lysates were run in non-reducing denaturing conditions 24 h later. Red asterisk marked lane 3 showed slow-migrating large size aggregates. (I) Same as H, except along with 22-FuG1, 6.30-FuG1 and a control non-target (a blood thinner drug called Pradaxa) binding Idarucizumab-FuG1 was used. Red asterisk marked lanes 2, 4, and showed slow-migrating large size aggregates. (J) Same as (E and F), except multiple additional controls were used including Idarucizumab-FuG1 during spike bearing pseudoviral generation. (K to L) Same as (E, F, and J) except in two independent biological experiments 4-fold and 6-fold concentrated lysates were loaded on the gel in case of pseudoviral generated in the presence of FuG1 antibodies. VSV-M is loading control, and each immunoblot is representative of two or three sets of experiments.

To test FuG1 mediated interference in generating fusion ready S2 fragment, multiple spike RBD and non-RBD targeting antibodies were tested (Fig. 5A, top). We hypothesized endosomal and surface accumulation of spike targeting antibodies before optimal spike expression and shutting to surface upon antibody treatments. HEK-293 cells were treated with 22-FuG1 and other control antibodies 4 h post-wild-type (WT) spike (S0) plasmid transfection. A control plasmid with mutations at the spike’s S1/S2 furin site (ΔRRAR) was also transfected to confirm the loss of furin cleavage (Fig. 5A, middle). At 24 h posttransfection (20 h antibody treatment), lysates were analyzed for ∼80KDa S2 fragment generation. FuG1 antibody was as effective as ΔRRAR mutant spike (6) in inhibiting spike cleavage at S1/S2 (Fig. 5B). Both 22-IgG1 and 22-IgG1 having random charge control (22-IgG1-RCC, see Fig. 5A bottom) distribution in Fc-extended peptides were ineffective. Similar results were evident in ACE2 receptor-expressing HEK-293 (293-ACE2) cells (Fig. 5C), VERO-E6 cells and calu3 cells (Fig. 5D). Next, we made use of previously described (35) ACE-2-RBD neutralizing (6.29-IgG1, 6.30-IgG1) and non-neutralizing (outside RBD region targeting, 12.19-IgG1, 12.25-IgG1) antibodies (Fig. 5A) (35). When 6.29, 6.30, 12.19, and 12.25 converted in FuG1 antibodies, similar spike S1/S2 cleavage inhibition was observed (Fig. 5E to G). These results were suggestive of the broad applicability of the FuG1 strategy. We must note that despite being non-inhibitory on spike S1/S2 cleavage, RBD neutralizing 6.29-IgG1 and 6.30-antibodies were significantly more effective than non-neutralizing RBD binding 22-IgG1, and non-RBD binding 12.25-IgG1, and 12.19-IgG1 antibodies in syncytia blockade (Fig. 5H). As syncytia formation requires fusion of spike expressing cells with other spike non-expressing (or expressing) ACE2^+^ cells, the latter supports the potential implication of the FuG1 strategy on the spike expression only in viral-producing cells (see Fig. 6, and 9). On the other hand, RBD neutralizing antibodies interfere at the interface of spike-ACE binding (35, 36). Nonetheless, the conversion of both RBD neutralizing and non-neutralizing antibodies (35) into FuG1 further enhanced their syncytia blockade function. However, it was significantly more evident for non-RBD neutralizing antibodies (Fig. 5H). We saw similar results of syncytia blockade, and reduced surface spike expression in VERO-E6 cells in the presence of 22-FuG1 antibody (Fig. 5I and J).

### Pseudovirions generated in the presence of FuG1 antibody contains inadequately processed spike.

Replication-restricted VSV particles (G*ΔG-luciferase-rVSV) harboring SARS-CoV-2 spike protein (pseudovirions) have been established to faithfully reflect the key aspects of host cell entry and infection (4, 5, 37–39). Thus, to test FuG1 mediated blockade of viral infections, we generated replication-defective WT and furin mutant (RRAR-GSAA: ΔRRAR) spike expressing luciferase-VSV pseudovirions named rVSV-spike*ΔG (WT-spike-rVSV) and rVSV-ΔRRAR-spike*ΔG (ΔRRAR-spike-rVSV) (Fig. 6A), without any prior antibody treatments as described earlier (5, 6, 37, 38). Then, 48 h later, viral supernatants were collected, followed by ultracentrifugation in a sucrose-gradient solution (5, 6, 38). Using immunoblotting, we observed significantly reduced S2 fragment release (spike cleavage inhibition) on ΔRRAR-spike-rVSV pseudovirions compared with WT-spike-rVSV (Fig. 6B) as described earlier (6). VSV-M served as loading control for equal viral lysates in these studies (Fig. 6).

Next, we tested replication-restricted WT spike-bearing luciferase-VSV pseudovirions (WT-spike-rVSV) in an established assay that uses luciferase readout values as an indicator of host cell entry (4, 5). WT-spike-rVSV pseudovirions were generated without prior treatment of antibodies (as described in Fig. 6B), followed by 1 h incubation with 22-IgG1 and 22-FuG1 (100 μg each), followed by transduction onto 293-ACE2 cells in 96-well plates. After 14 to 16 hr post-transduction, lysates were analyzed for luciferase activity as described earlier (5). 22-FuG1 was only marginally more effective over 22-IgG1, although insignificant (Fig. 6C). These results indicated that a significant S1/S2 furin cleavage event occurs in spike expressing (and viral producing cells) prior to spike incorporation on replication-defective VSV particles (Fig. 9A). The reduction in luciferase activity (Fig. 6C) by both 22-IgG1 and 22-FuG1 is potentially due to their RBD binding partial neutralization function, as shown earlier (40). Next, pseudovirions were generated in the presence of FuG1 antibodies (Fig. 6D). To this end, BHK1 (or 293-T) cells were pretreated with IgG1 control, 22-IgG1, 22-FuG1, and 22-IgG1-RCC antibodies 4 h post-spike (S0) transfection (see Fig. 5). Cells were re-treated 24 h later with corresponding antibodies, and replication-restricted G*ΔG-luciferase-rVSV particles were added on to cells (38). Harvested viral supernatants were analyzed 48 h later for S0 and S2 fragments using ultracentrifugation followed by immunoblotting where VSV-M protein served as pseudoviral loading control (Fig. 6E). Unexpectedly, 22-FuG1 significantly destabilized pseudoviral spike (S0) along with an almost complete reduction in fusion ready S2 fragment generation while both 22-IgG1, 22-IgG1-RCC were largely ineffective (Fig. 6E). Similar S0 destabilization was evident on spike-rVSV generated in the presence of 6.29-FuG1 and 6.30-FuG1 antibodies (Fig. 6F, see last two lanes). Thus, both target (spike) binding and furin interference function of FuG1 antibodies also potentially interferes with optimal particle incorporation and spike (S0) destabilization in virus-producing cells.

### Spike (S0) destabilization by FuG1 strategy.

Interestingly, similar to destabilized pseudoviral spike (S0), a significantly lower levels of full-length S0 protein expression were also evident in spike-targeting FuG1 antibody treated cellular lysates (Fig. 5B to G, see S0 band). The latter was despite the similar level of DNA concentrations during spike plasmid transfection. These results collectively suggested additional FuG1-mediated structural destabilization/degradation of full-length S0 spike potentially due to sustained and non-cleavable interactions of furin-FuG1-spike in the tripartite complex. Because the spike transfected cellular lysates were made within 16 to 20 h of various antibody treatments (Fig. 5B to G), to reassess potential protein destabilization of the spike (S0) protein, we increased the treatment time (>48 h) of FuG1 antibodies before generating cellular lysates. Strikingly, along with inhibition of S1/S2 cleavage, we also observed a time-dependent reduction in spike S0 levels (Fig. 6G).

Recently, spike RBD targeting and *in silico* engineered Fc-fusions peptides demonstrated robust proteasome-directed spike degradation to inhibit viral loading and production (41). Furthermore, the large protein aggregates are routinely fragmented and processed by proteasome complex (42, 43). Because the Fc-fusion-based FuG1 strategy also reduces the overall spike (S0) levels (both cellular transfections and pseudovirions, Fig. 5B to G, Fig. 6E, F), we tested the possibility of generation of higher size aggregate complexes on the SDS gel immunoblots. Furthermore, as FuG1 is bivalent, its interactions with more than one tripartite spike-FuG1-furin complex to generate higher size aggregates is a potential possibility for spike destabilization and reduced overall S0 levels in a proteasome-dependent manner. To this end, we carried out non-reducing denaturing gels of FuG1 and other control antibody-treated samples followed by spike immunoblotting. Notably, only FuG1 treated WT spike transfected cellular lysates showed higher aggregated complexes with immunoblotting signal against spike antibody (Fig. 6H, third lane with red asterisk). Similar results were evident with another FuG1 (6.30-FuG1) antibody-treated against WT spike transfected cells (Fig. 6I). Strikingly in both the scenarios, the ΔRRAR mutant spike transfected cells did not generate these higher aggregated complexes upon FuG1 treatments (Fig. 6H and I). Thus, simultaneous FuG1’s variable domain (Fab) interactions with spike RBD, coupled with Fc-peptide interactions with spike-furin complex, are critical for spike (S0) instability.

To confirm the potential role of the tripartite spike-FuG1-furin complexes being responsible for the higher aggregate formation and S0 destabilization, we made use of a non-cellular target (Pradaxa drug) engaging idarucizumab-FuG1 antibody (also see Fig. 4L). Because BHK1 cells (or MEM media) or endosomes do not express or contain blood thinner Pradaxa, we hypothesized that because of lack of spike target binding, the idarucizumab-FuG1 would not destabilize S0. No higher spike aggregates were evident in idarucizumab-FuG1 treated lysates (Fig. 6I, compare lane 3 with 2, 4, and 5 with a red asterisk on top of gel). When tested, despite similar levels of expression viral assembly matrix protein (VSV-M), significantly reduced levels of spike S0 were loaded on pseudovirions only when generated in the presence of 22-FuG1 antibody but not idarucizumab-FuG1 (Fig. 6J, lanes 5, 6 versus 7, 8). When 4× and 6× pseudoviral lysates were loaded (evident with VSV-M protein), full-length S0 protein levels were similar to untreated and IgG1 treated samples (Fig. 6K). Furthermore, even in these conditions, FuG1 antibodies significantly inhibited cleavage at S1/S2 sites, as evident with highly reduced S2 signal (Fig. 6K, compare lanes 1 to 4 vs 5, 6). We reconfirmed these results with the second set of assays in similar conditions (Fig. 6L). Collectively, spike-targeting FuG1 strategy not only competitively inhibits furin S1/S2 cleavage function but also interferes with viral particle incorporation in infected cells via destabilizing S0 levels (Fig. 9, working model).

### Proteasome inhibition restores spike S0 levels in FuG1 treated lysates and pseudovirions.

**FIG 7:**
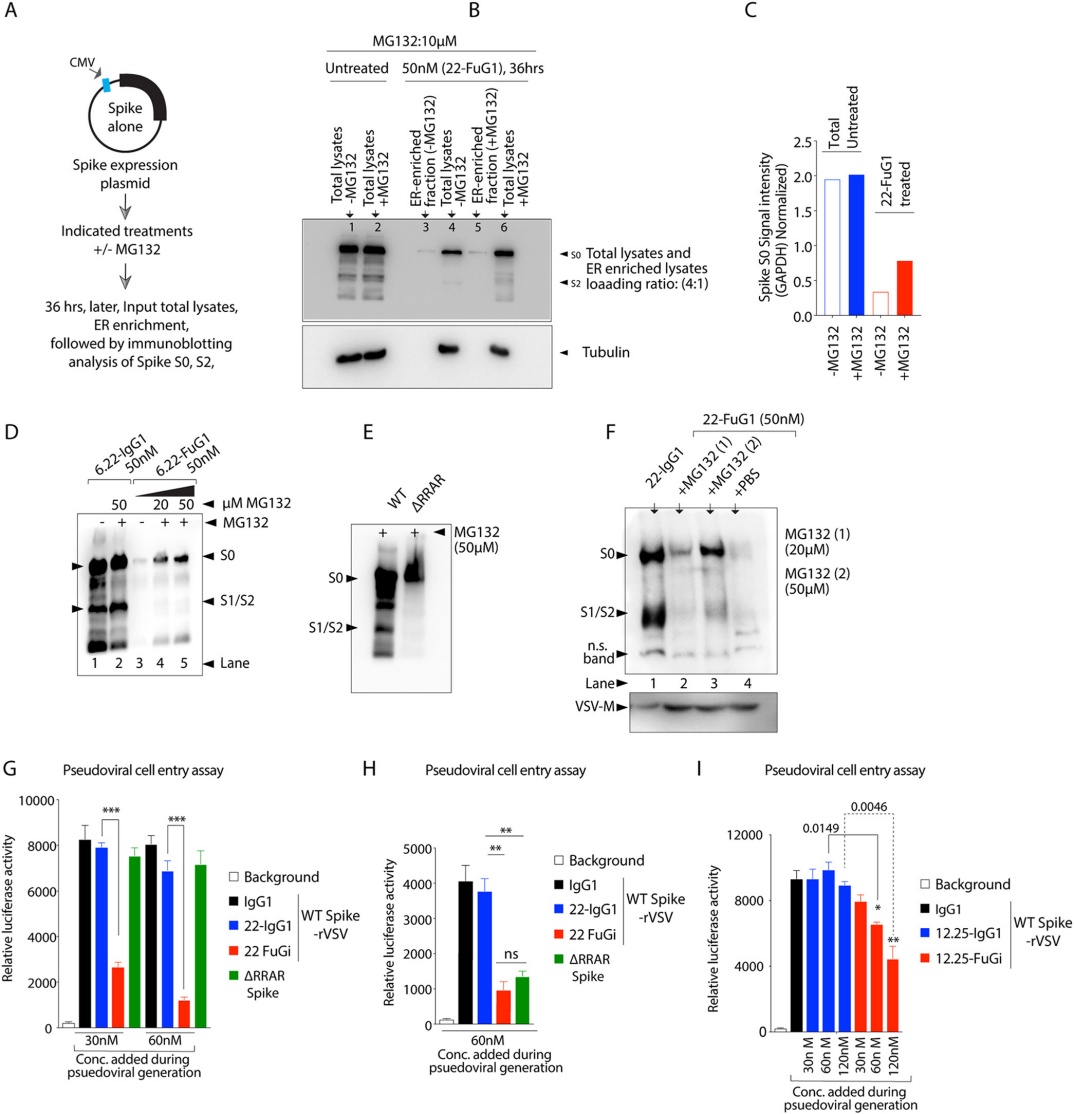
Spike S0 destabilization by FuG1 strategy is proteasome dependent. (A) Workflow of data shown in B. (B) Spike transfected, non-FuG1 treated (lanes 1, 2) and spike transfected plus 22-FuG1 treated (lanes 4, 6) were subjected to immunoblotting analysis of spike ±MG-132 (10 μM). Lanes 3 and 5 were loaded with control ER-enriched lysates (5 μg) to confirm if equal amount of translating spike protein was present ±MG-132 proteasome inhibitor in FuG1 treated samples. (C) Spike, S0 signal intensity in total lysates (±MG-132 treated) after normalization with GAPDH is shown. (D) 4 h post-spike DNA transfection, indicated antibodies (on top) were added onto the 293-ACE2 cells ± increasing (20 μM and 50 μM) MG-132 concentrations. Lysates were prepared 36 h posttransfection following by immunoblotting against spike. (E) Experimental control to confirm that MG-132 treatment does not interfere with WT spike processing and non-processing of ΔRRAR spike. (F) WT spike transfected BHK1 cells were pre-treated with indicated antibodies (± 20 μM and 50 μM MG-132 or PBS alone) after 4 h. 12 h later replication-restricted G*DG-luciferase-rVSV particles were added on to the cells. 48 h later harvested pseudovirions lysates were analyzed for S0 and S2 fragments using immunoblotting on viral particles. VSV-M and a nonspecific band in spike blot is loading control. (G to I) Same as Fig. 6E, F, J, K, L, except spike bearing pseudoviral particles harvested in the presence of 22-IgG1 and 22-FuG1 antibodies were transduced onto 293-ACE2 cells (TMPRSS2- cells in G and I) and Calu-3 cells (TMPRSS2^+^ cells in H). After 24 h, virus-encoded luciferase signal was quantitated from the lysates as a measure of host cell entry. ΔRRAR pseudoviral particles were used as control for both TMPRSS2- and TMPRSS2^+^ cells as described earlier (5). Error bars indicates SEM (*n* = 3). Statistical significance in G-I was defined by unpaired T-test with Welch’s correction (*, *P* < 0.05; **, *P* < 0.005; ***, *P* < 0.0001) and immunoblots are representative of two or three independent experiments.

Similar to the published findings of proteasome function mediated spike destabilization by *in silico* engineered Fc-fusion peptides (41), we observed partial rescue of spike S0 loss at 10 μM concentration of proteasome inhibitor concentration MG132 (Fig. 7A and B). When analyzed via subcellular fractionation, preliminary spike S0 signal in ER enriched fraction was very similar, while total spike S0 signal was at least 2-fold higher in FuG1 and MG132 treated lysates (Fig. 7B and C). To further confirm these findings, we increased MG132 concentrations (Fig. 7D). Treatment of 50 μM MG132 significantly increased total spike S0 signal, while S1/S2 cleaved fragment was almost invisible (Fig. 7D, compare lanes 2 and 3 versus lanes 2 and 5). As expected, MG132 alone did not interfere with the furin processing and non-processing of WT and ΔRRAR spike, respectively (Fig. 7E). To further confirm these results, we generated pseudovirions in the presence of FuG1±MG132 similar to the conditions described earlier (Fig. 6E, F, J to L). When harvested viral supernatants were analyzed for S0, and S2 fragments (48 h later), MG132 treatment rescued spike S0 destabilization in FuG1 treated samples (Fig. 7F, compare lanes, 2, 3 vs 4). Notably, as expected, S2 fragment generation remained inhibited despite S0 stabilization (Fig. 7F). These results further strengthen the dual working mechanism of the FuG1 strategy to competitively inhibits furin S1/S2 cleavage function and S0 destabilization. Thus, both altered spike confirmation around the S1/S2 region (6) and potentially large aggregates of spike-FuG1-furin interferes with optimal viral transmembrane coat protein assembly.

Next, using 293-ACE2 cells, we tested the effect of reduced S1/S2 cleaved and reduced pseudoviral loaded spike in transduction assays. With luciferase activity as a measure of viral pseudo-infection (host cell entry), WT-spike-rVSV pseudovirions generated in the presence of 22-FuG1 (with increasing concentrations) were significantly less infectious compared to ΔRRAR-spike-rVSV pseudovirions after 24 h (Fig. 7G). In support of previous studies, furin mutation did not change cellular entry of ΔRRAR-spike-rVSV mutant pseudovirions (5). It must be noted that, unlike 22-FuG1, WT-spike-rVSV pseudovirions generated in the presence of 22-IgG1 gained cellular entry very similar to pseudovirions generated with IgG1 control treatment. Similar results of significantly reduced host cell entry (viral infection) were evident when WT-spike-rVSV pseudovirions were generated in the presence of other spike targeting antibodies converted into FuG1 antibodies and were tested using TMPRSS2- 293-ACE2cells (Fig. 7I). Importantly, in support of previous studies (5), ΔRRAR-spike-rVSV mutant pseudovirions remained limitedly infectious against TMPRSS2^+^ Calu-3 cells (Fig. 7H). These results additionally support the furin inhibition mediated gain of S0 destabilization and S1/S2 cleavage interference function of FuG1 strategy, which collectively interfere with optimal particle incorporation in infectious particle producing cells during viral egress and subsequent infections (6).

### Spike targeting by FuG1 provides furin inhibition specificity.

**FIG 8:**
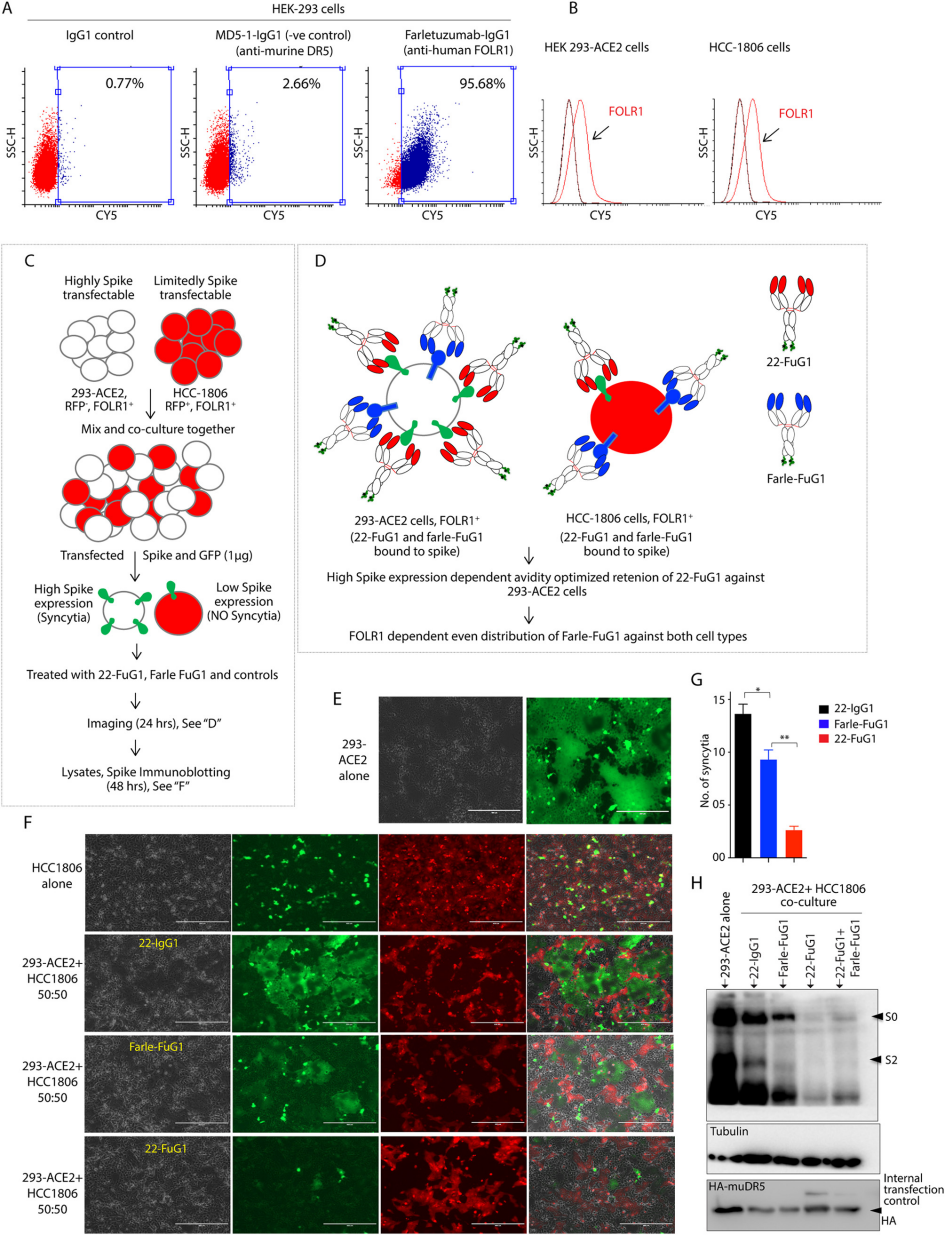
Selectivity of FuG1 strategy against spike expressing cells. (A) Flow cytometry analysis of surface FOLR1 in HEK-293 cells. (B) Flow cytometry confirmation of similar level of FOLR1 expression in 293-ACE and HCC-1806 cells. (C) Schematic of the coculture experiment described in E and F. (D) Schematic showing preferred binding of 22-FuG1 (red) toward high spike expressing 293-ACE2 cells, while farletuzumab-FuG1 (Blue) distributes equally against both cell lines expressing similar levels of FOLR1 (see B). (E, F) Cocultured cells were treated with 22-IgG1, farletuzumab-FuG1, and 22-FuG1 antibodies 4 h after spike-EGFP construct transfection. Cells were imaged 30 h later at ×10 magnification using Evos fluorescence microscope. (G) Number of syncytia from F. Error bar indicated SEM (*n* = 3). (H) Same experiment as E to G, except additional internal transfection control HA-tagged murine-DR5 (HA-muDR5-pCDNA3.1) was co-transfected with spike-EGFP construct. Total lysates from spike-transfected 293-ACE2 cells alone, and cocultured-cells, were subjected to lysate preparation 48 h later, followed by immunoblotting analysis of S0 and S2. HA-muDR5 served an internally equivalent transfection efficiency control and tubulin is lysate loading control. Statistical significance in G was defined by unpaired T-test (*, *P* < 0.05; **, *P* < 0.005).

To confirm the FuG1 approach’s specificity of furin blockade only against spike-transfected cells, we cocultured a comparable level of FOLR1 expressing (Fig. 8A and B) 293-ACE2 and RFP-stable HCC-1806 cells (Fig. 8C). Previous studies have established selective detection of only multiple-nucleated enlarged syncytium at low fluorescent magnification (10×) if the spike protein was transfected along with GFP (44). As a result, the GFP signal is almost undetectable from non-syncytial mononucleated cells at <×10 magnification (44). Spike-EGFP transfected cell cocultures were treated with 100 μg 22-IgG1, farletuzumab-FuG1, and 22-FuG1 antibodies in a manner similar to that described in Fig. 5. The HCC-1806 cells are limitedly transfectable compared with 293-ACE2, <20% vs >85% based on pcDNA3.1-EGFP alone transgene expression (data not shown). Thus, we hypothesized equal FOLR1 expression dependent early (or late) endosomal/lysosomal or cell surface occupancy of farletuzumab-FuG1 against both cell-types in cocultures (Fig. 8D). On the contrary, 22-FuG1 would exclusively target and enrich early (or late) endosomal/lysosomal or cell surface compartments against high spike expressing 293-ACE2 cells. The broad distribution of farletuzumab-FuG1 in the coculture assays would inhibit furin function regardless of spike expression. On the contrary, 22-FuG1 would selectively inhibit furin-mediated spike cleavage in target (spike) expressing cells. When tested, farletuzumab-FuG1 only reduced ∼30% syncytia load compared to 22-FuG1 (∼90%) in cocultures (Fig. 8E to G). Although we expected significantly more syncytia (60% to 70% vs ∼30%) in farletuzumab-FuG1 treated conditions, the discrepancy could be potentially due to higher cellular stability and recycling function of farletuzumab antibody, which, unlike 22-IgG1, is a humanized clinical antibody with optimal serum stability (45). Regardless 22-FuG1 was significantly more effective (Fig. 8G). When various antibody-treated and coculture cell-generated lysates were analyzed after 48 h of treatment, farletuzumab-FuG1 only partially reduced S2 conversion in lysates, while 22-FuG1 was highly effective in reducing both S0 and S2 (Fig. 8H). These results collectively support cellular selectivity toward furin inhibition (and interference with S2 generation) and potential spike destabilization by the FuG1 strategy.

## DISCUSSION

**FIG 9:**
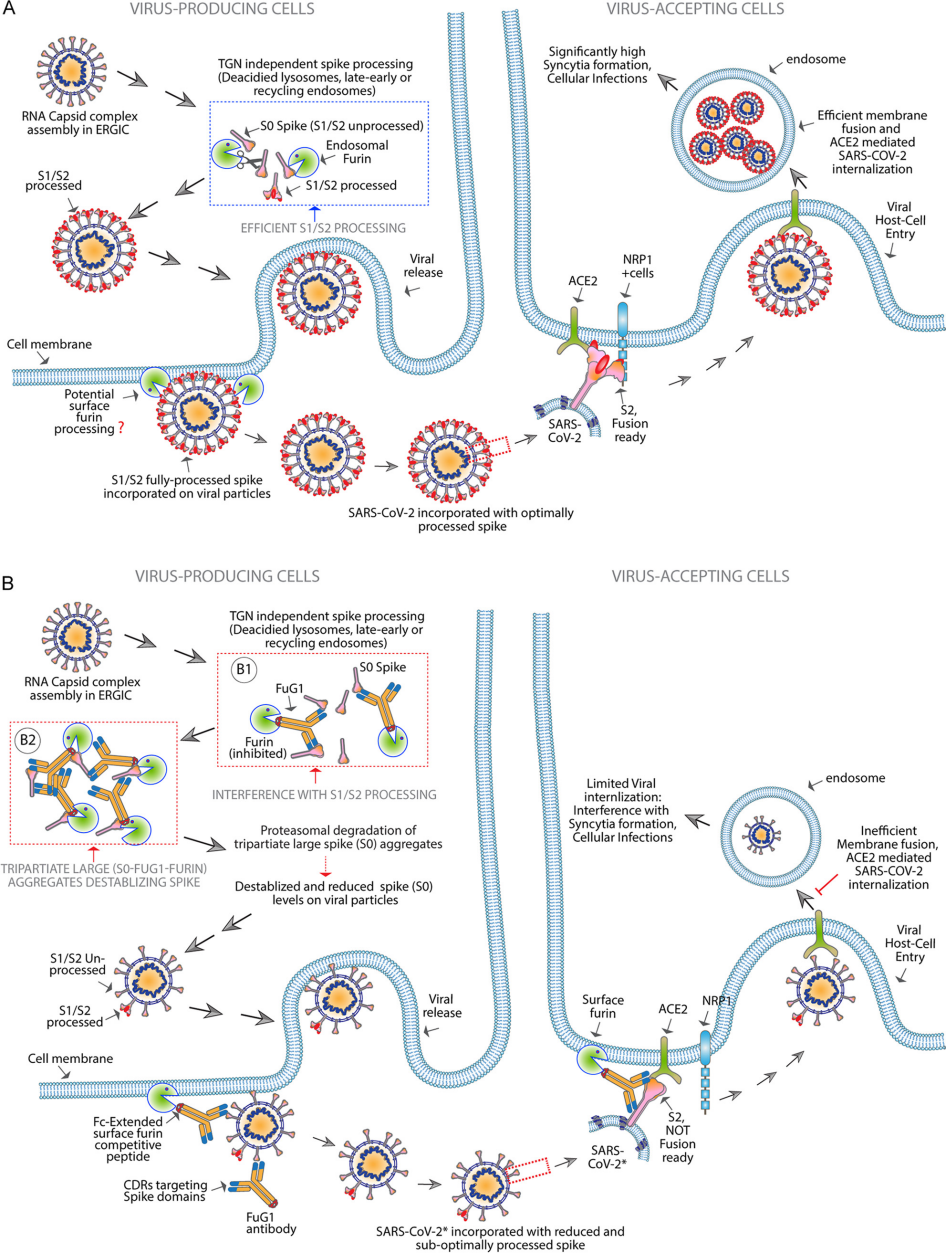
Working mechanism of FuG1 strategy. (A) In SARS-CoV-2 infected (viral replicating) cells, after RNA packing and viral structural assembly in endoplasmic-reticulum Golgi-intermediate compartment (ERGIC), the spike harboring RNA capsid assembly complex is shuttled toward deacidified lysosomal, late, early, recycling or membrane endosomes route (12) rather than TGN secretory pathway (see Fig. 3). The endosomal furin mediates proteolytic processing of spike S1/S2 before viral release. If cell surface enriched furin also contributes to spike cleavage could not be ruled out. Nonetheless, furin cleavage generates fusion-ready S2 fragments, allowing spike to bind NRP1(4) effectively to enhance overall viral cellular entry in ACE2+ acceptor cells. (B) Membrane and endosomal recycling and spike targeting FuG1 antibodies make use of spike binding as an anchor to competitively disrupt furin proteolytic processing of spike S1/S2 in the membrane-endosomal network. Furthermore, large size complex aggregates (slower migrating on gel) are generated potentially due to tripartite SARS-CoV-2 spike-FuG1-furin multivalent interactions (see Fig. 6). The resulting higher-order aggregates are degraded by proteasome complex (Fig. 7) along with potentially additional unknown mechanisms. The latter collectively reduces and destabilizes overall S0 levels on released viral particles (Fig. 5 and 6). All together, both suppression of S0 to S2 generation (B1) and S0 destabilization (B2) limit spike particle incorporation and subsequent viral entry and infection in ACE2^+^ acceptor cells.

Here we describe a targeted antibody-based plug-and-play FuG1 strategy that not only directly interferes with the furin-dependent proteolytic mechanism of spike cleavage and activation but also destabilizes the full-length spike protein. Both mechanisms collectively interfere with the optimal spike incorporation during viral assembly in producing cells with the potential to impede with SARS-CoV-2 chain of infection cycle (Fig. 9). Considering that externally provided antibody-based targeting of intracellular proteins (such as those residing in the cytosol, ER, or TGN network) is nearly impossible (46), FuG1 strategy-mediated targeting of furin likely operates outside TGN. Nonetheless, the regulatory role of furin in the endosomal network and cell surface is well established (28). Importantly, both endosomes and cell membranes are a well-defined route of therapeutic antibodies in complex with target-antigen or neonatal Fc receptor (Fc-Rn) based recycling mechanisms (13, 47). Thus, FuG1 strategy-mediated blockade of furin-driven spike S1/S2 cleavage in the endosomal network of viral-producing cells is consistent with published studies (12, 48). Furthermore, the described organelle fractionation studies additionally strengthen the furin-mediated spike processing and viral egress via the deacidified lysosomal/endosomal system (12). While it is possible that lysosomal deacidification may be due to the overloading of highly glycosylated spike protein or perturbation of ion-channel pumps in the membrane (48), it remains to be demonstrated. Furthermore, although our results remain limited to ectopic spike expression and spike-harboring pseudovirions, additional testing with authentic SARS-CoV-2 is needed.

Spike-rVSV particles generated even in the presence of furin inhibiting FuG1 antibodies were able to maintain close to ∼15% to 40% pseudo-infection (depending on the spike targeting FuG1 used), as evident with the host cell entry assays (Fig. 7G to I). The latter could be attributed to multiple independent working mechanisms. First, FuG1 antibodies did not completely inhibit pseudoviral S2 generation (Fig. 6 and 8), which demands further optimization of the FuG1 peptide. Second, various SARS-CoV-2 neutralizing, or non-neutralizing antibodies fall into multiple categories based on their interactions with spike RBD or non-RBD regions (13). For example, despite no effect on spike S0 to S2 conversion, unlike 12.25 IgG1, 12.19 IgG1, and 22-IgGI antibodies, core-RBD targeting 6.30 or 6.29-IgG1 antibodies (40) were significantly effective in syncytia blockade (Fig. 5H). On the other hand, 22-FuG1 was as effective as 6.30 or 6.29-FuG1 antibodies in suppressing spike S0 to S2 cleavage. Thus, the disparity in syncytia blockade is not an optimal predictor of an effective IgG1 conversion into FuG1. In addition, the RBD domain of spike undergoes hinge-like movements in “up” “down” conformations, representing ACE2-accessible or inaccessible states, respectively (49, 50). Considering the unpredictable interactions of SARS-CoV-2 neutralizing and non-neutralizing antibodies with RBD up and down confirmations (13, 49), defining a particular epitope on the spike for maximum accessibility of FuG1 peptide to competitively saturate furin at “RRAR” sites is critical in improving the targeting strategy further (3, 50, 51).

Third, previous reports (39) have also described the potential furin independent spike priming by cysteine proteases such as cathepsin B/L (CatB/L) for aiding the infection in potential TMPRSS2^-^ cells. In support, S1/S2 mutant ΔRRAR pseudoviral rVSV-spike particles gained effective entry in the transduction experiment in TMPRSS2- cells but not in TMPRSS2^+^ cells (Fig. 7G to I). Considering that the natural SARS-CoV-2 virus with furin mutations was partially infectious in murine models (6), the combinatorial targeting of FuG1 with TMPRSS2 and CatB/L inhibitors is expected to weaken the viral pathogenicity further. Regardless, additional testing is needed in relevant infectious animal models with authentic SARS-CoV-2.

Other than furin-targeting small molecular inhibitors, several studies have described engineered ACE2 trap (8) and mini protein-based peptide designs (9) to neutralize SARS-CoV-2 infection. Unfortunately, many of these approaches lack targeting specificity against the viral-infected cell and are highly likely to inhibit cellular protease randomly to induce toxicity (10). On the other hand, the described FuG1 approach potentially gives spike target mediated specificity over-described furin/TMPRSS2 protease network targeting small molecule inhibitors and protein-based designs (7–9). Furthermore, dual-specific antibody-based FuG1 strategies co-targeting lung-enriched antigens that offer enhanced specificity to infected lung cells and tissue could be engineered (20). Besides, as the FuG1 approach is only dependent on exploiting spike epitope as a targeting anchor (Fig. 9), if the target antibody's epitope is not affected by naturally evolving mutations in SARS-CoV-2 variants (Delta, Omicron, etc.), the strategy could potentially be broadly applicable (2, 52). However, testing relevant COVID-19 infectious models remains critical in establishing the therapeutic applications of FuG1-strategy against SARS-CoV-2 variants (6). Nonetheless, the FuG1 strategy has a crucial advantage over other protease targeting strategies in specificity.

Interestingly, earlier reports have described higher incorporation of ΔRRAR mutant spike glycoprotein (higher particular/PFU ratio) on the infectious SARS-CoV-2 virus than WT spike (6). Strikingly, FuG1-targeted and furin competing WT spike showed reduced stability and particle incorporation. Our results provide evidence for the observed disparity of viral incorporation between ΔRRAR mutant spike versus FuG1-targeted WT spike glycoprotein. Unlike WT spike, ΔRRAR mutant spike is not a furin substrate (6) and does not generate slow migrating higher size FuG1-spike-furin aggregates (Fig. 6H and I). In contrast, WT spike induces higher size FuG1-spike-furin aggregates. Hypothetically, the latter might be due to the lower furin dissociation rate of FuG1 (Fig. 2C), resulting in the formation of larger multivalent tripartite spike-FuG1-furin complexes. In supporting previous studies (41), such aggregates destabilize spike protein in a proteasome-dependent function. Nevertheless, our results support the published studies of spike destabilization by Fc-fusion peptides and furin-mediated events critical for SAR-S-CoV-2 pathogenesis upon cellular entry (6, 41).

In summary, the FuG1 approach represents rational spike biology and processing-based plug-n-play Fc-extended peptide design to target the SARS-CoV-2 chain of infection function. With further testing of authentic and infectious SARS-CoV-2, the FuG1 could be an alternative therapeutic strategy against the emerging coronaviridae variants that exploit cellular proteases for viral entry and deadly infections.

## MATERIALS AND METHODS

### Source of antibody sequences.

The sequence of anti-SARS-CoV spike RBD (RBD-C) targeting immunoglobulin heavy chain and light chain are available at GenBank: DQ168569.1 (CR3022 VH) and GenBank: DQ168570.1 (CR3022 VL). The sequence of anti-SARS-CoV spike (outside RBD, named S-B) targeting immunoglobulin heavy chain and light chain are available at GenBank: MT594062.1 (12.25 VH) GenBank: MT594095.1(12.25 VL). These antibodies are described previously (35). The sequence of farletuzumab, KMTR2, etc. used are previously described (20) and are publicly available at imgt.org.

### Recombinant antibody cloning.

The sequence sources of various spike-targeting antibodies used in this study is provided. Additional antibodies such as farletuzumab, KMTR2, avelumab, idarucizumab clones has been published by our research group and are described earlier (20, 34, 53). Generation of linkered IgG1 has been described earlier by our group, see Fig. 1 in reference (20). Briefly, the DNA sequences were retrieved from the open sources (IMGT.ORG or publicly available patents or NCBI etc.) and synthesized as gene string with overlapping region to pCDNA3.1 vector using Invitrogen GeneArt gene synthesis services. Using overlapping pCDNA3.1 restriction site EcoRI and HindIII primers, PCR amplification was carried out of gene VH and VL gene strings separately. After PCR amplification, DNA was gel purified and inserted into pCDNA 3.1 vectors (CMV promoter) by making use of In-Fusion HD Cloning Kits (TaKaRa Bio). EcoRI and HindIII digested vector was incubated with overlapping PCR fragments (of various different recombinant DNA strings of antibodies) with infusion enzyme (1:2, vector: insert ratio) at 55°C for 30 min, followed by additional 30-min incubation on ice after adding Escherichia coli Stellar cells (Clontech). Transformation and bacterial screening were carried out using standard cloning methods. Positive clones were sequenced confirmed in a three-tier method. Confirmed bacterial colonies were Sanger sequenced upon PCR followed by re-sequencing of mini-prep DNA extracted from the positive colonies. Finally, maxiprep were re-sequenced prior to each transfection. Recombinant antibodies were also re-confirmed by ELISA and flow cytometry surface binding studies as described earlier (20). FuG1 antibodies were also engineered in similar way and the detailed sequence of FuG1 Fc-extended peptides is provided in the manuscript figures. Linkered monospecific and bispecific with knob and hole mutations were generated using flexible linkers as described previously (20).

### Recombinant RBD IgG4-Fc cloning.

To clone spike RBD domain, amino acids 333 to 530 were ordered for gene string synthesis in continuation of G4S flexible linker and IgG4 CH2 and CH3 domain with EcoRI and HindIII overlapping region. Following PCR amplification with overlapping primers, DNA was gel purified and inserted into pCDNA 3.1 vectors (CMV promoter) by making use of In-Fusion HD Cloning Kits (TaKaRa Bio) as described in previous section. Recombinant IgG4-Fc FOLR1 and DR5 were also cloned in similar manner and have been described previously (20).

### Recombinant antibody and IgG4-Fc DR5, RBD, FOLR1 expression.

Free style CHO-S cells (Invitrogen) were cultured and maintained according to supplier’s recommendations (Life technologies) biologics using free style CHO expression system (life technologies) and as previously described (20, 54). A ratio of 1:2 (light chain, VL: heavy chain, VH) DNA was transfected using 1 μg/mL polyethyleniamine (PEI). After transfection cells were kept at 37°C for 24 h. After 24 h, transfected cells were shifted to 32°C to slow down the growth for 9 additional days. Cells were routinely feed (every second day) with 1:1 ratio of Tryptone feed and CHO Feed B. After 10 days, supernatant from cultures was harvested and antibodies were purified using protein-A affinity columns. Various recombinant antibodies used in this study and recombinant target antigens were engineered, expressed, and purified in Singh Laboratory of Novel Biologics as described earlier (20). Recombinant antigens were similar expressed and transfected with PEI at 1 μg/mL concentration. All purified proteins were also confirmed for size using reducing SDS-PAGE, and standard ELISA as described earlier (20).

### Antibody purification.

Various transfected IgG1 antibodies, FuG1 antibodies, and recombinant IgG4-Fc antigens (as indicated in text and figure legends) were affinity purified using HiTrap MabSelect SuRe (GE, 11003493) protein-A columns. Transfected cultures were harvested after 10 days and filtered through 0.2-micron PES membrane filters (Milipore Express Plus). Cleaning-in-place (CIP) was performed for each column using 0.2M NaOH wash (20 min). Following cleaning, columns were washed three times with Binding buffer (20 mM sodium phosphate, 0.15 M NaCl, pH 7.2). Filtered supernatant containing recombinant antibodies or antigens were passed through the columns at 4°C. Prior to elution in 0.1M sodium citrate, pH 3.0 to 3.6, the columns were washed three times with binding buffer (pH 7.0). The pH of eluted antibodies was immediately neutralized using sodium acetate (3M, pH 9.0). After protein measurements at 280 nm, antibodies were dialyzed in phosphate-buffered saline (PBS) using Slide-A-Lyzer 3.5K (Thermo Scientific, 66330). Antibodies were run on gel filtration columns (see next section) to analyze the percent monomers. Whenever necessary a second step size exclusion chromatography (SEC) was performed. Recombinants IgG4-Fc tagged extracellular domain antigens such as rFOLR1, rDR5, and RBD, etc. were also similarly harvested and purified using protein-A columns.

### Size exclusion chromatography.

The percent monomer of purified antibodies was determined by size exclusion chromatography. 0.1 mg of purified antibody was injected into the AKTA protein purification system (GE Healthcare Life Sciences) and protein fractions were separated using a Superdex 200 10/300 column (GE Healthcare Life Sciences) with 50 mM Tris (pH 7.5) and 150 mM NaCl. The elution profile was exported as Excel file and chromatogram was developed. The protein sizes were determined by comparing the elution profile with the gel filtration standard (Bio-Rad 151–1901) (55). Any protein peak observed in void fraction was considered as antibody aggregate. The area under the curve was calculated for each peak and a relative percent monomer fraction was determined as described earlier (20).

### Binding studies by ELISA.

Binding specificity and affinity of various described IgG1’s and FuG1 antibodies (including linkered FuG1, FuG1-Lin) s were determined by ELISA using the recombinant extracellular domain of corresponding receptor/target antigen. For coating 96-well ELISA plates (Olympus), the protein solutions (2 μg/mL) were prepared in coating buffer (100 mM Sodium Bicarbonate pH 9.2) and 100 μL was distributed in each well. The plates were then incubated overnight at 4°C. The next day, the unbound areas were blocked by cell culture media containing 10% FBS, 1% BSA, and 0.5% sodium azide for 2 h at room temperature. The serial dilutions of antibodies (2-fold dilution from 50 nM to 0.048 nM) were prepared in blocking solution and incubated in target protein coated plates for 1 h at 37°C. After washing with PBS solution containing 0.1% Tween20, the plates were incubated for 1 h with horseradish peroxidase-(HRP) conjugated anti-human IgG1 (Thermo Scientific, A10648). Detection was performed using a two-component peroxidase substrate kit (BD Biosciences) and the reaction was stopped with the addition of 2N Sulfuric acid. Absorbance at 450 nm was immediately recorded using a Synergy Spectrophotometer (BioTech), and background absorbance from negative control samples was subtracted. The antibody affinities (KD) were calculated by non-linear regression analysis using GraphPad Prism software.

### Flow cytometry.

The cell surface expression of ACE2, spike, DR5, FOLR1, etc. was analyzed by flow cytometry in various experiments (see figure and figure legends) after treatment with various control and FuG1 antibodies. After various treatments, cells were trypsinized and suspended in FACS buffer (PBS containing 2% FBS). The single cell suspension was then incubated with primary antibodies for 1 h at 4°C with gentle mixing. Following wash with FACS buffer, the cells were then incubated with fluorescently labeled anti-Rabbit antibody for 1 h. Cells were washed and flow cytometry was performed using FACSCalibur. The data was analyzed by FCS Express (*de novo* software) and FlowJo.

### Binding studies by biolayer interferometry.

Binding measurements were performed using bio-layer interferometry (BLI) on FortéBio Red Octet 96 instrument (Pall) as described earlier (20). Biotin-Streptavidin based sensors were employed for the studies. Recombinant Fc linked DR5 variants were biotinylated using EZ-Link Sulfo-NHS-SS-Biotin (Thermo Scientific, 21331) following the manufacturer’s instructions. Unreacted sulfo-NHS-SS-biotin reaction was quenched by 50 mM Tris-Cl pH 7.4 and removed via dialyzing against PBS. For binding analysis biotinylated antigen (1 μg/mL) were immobilized on streptavidin (SA) biosensors (Pall) for 300 s to ensure saturation. Associate and dissociation reactions were set in 96-well microplates filled with 200 μL of unbiotinylated DR5 agonist for 900 s. All interactions were conducted at 37°C in PBS buffer containing 2 mg/mL BSA. These binding observations were also confirmed by biotinylating the agonist antibodies and probed against unbiotinylated DR5-Fc variants. Kinetic parameters (K_ON_ and K_OFF_) and affinities (KD) were analyzed using Octet data analysis software, version 9.0 (Pall).

### Furin protease activity assay.

Different concentrations of farletuzumab-FuGi, farletuzumab-cFuGi (25 to 250 ng), or furin assay buffer (negative control) were preincubated with 25 ng of furin (50 μL, 0.5 ng/μL in furin assay buffer) in a total reaction volume of 100 μL for 10 min at room temperature. The reaction was started by adding 40 μL furin protease substrate (final concentration of the furin protease substrate in a 100 μL reaction was 2 μM). The fluorescence intensity of the reaction mixture was monitored at excitation wavelength of 380 nm and detection of emission at wavelength 460 nm using 96-well microplate reader (BioTek Instruments Inc., USA). For inhibitor control, 10 μL of chloromethylketone (0.5 μM) was added to 25 ng of furin and for positive control (only furin), no antibody and inhibitor were added. The protease activity of positive control well was considering as 100% activity and the decrease in furin activity was compared to that. For kinetic study, fluorescence was measured immediately after adding the protease substrate and data was recorded every 1 min for 15 min at Ex/Em = 380 nm/460 nm.

### General western blotting.

Cells were cultured overnight in tissue culture-treated 6-well plates prior to treatment. After antibody treatment for 48 h (or indicated time), cells were rinsed with PBS and then lysed with RIPA buffer supplemented with protease inhibitor cocktail (Thermo Scientific). Spinning at 14,000 rpm for 30 min cleared Lysates and protein was quantified by Pierce BCA protein assay kit. Western blotting was performed using the Bio-Rad SDS-PAGE Gel system. Briefly, 30 μg of protein was resolved on 10% Bis-Tris gels and then transferred onto PVDF membrane. Membranes were blocked for 1 h at room temperature in TBS + 0.1% Tween (TBST) with 5% non-fat dry milk. Membranes were probed overnight at 4°C with primary antibodies. Membranes were washed three times in TBST and then incubated with anti-rabbit or anti-mouse secondary antibodies (1/10,000 dilution, coupled to peroxidase) for 1 h at room temperature. Membranes were then washed three times with TBST and immunocomplexes were detected with SuperSignal West Pico Chemiluminescent Substrate (Thermo Fisher Scientific). Images were taken using a Bio-Rad Gel Doc Imager system.

### Syncytia formation and inhibition studies in the presence of FuG1 antibodies.

293T cells expressing stable ACE2 (293-ACE2) have been described earlier (56) and were kindly provided Dr. Jesse D. Bloom. 293-ACE2 were transfected with 1 μg (unless mentioned) of spike-EGFP plasmid using mirus reagent as per manufacturer’s instructions. Cells were monitored for the formation of syncytia after 20 h of incubation. In case of syncytia inhibition experiment, various control and FuG1 antibodies were added with indicated concentrations (see figures and figure legends for specific concentration), 4 h after transfection. For some experiments, such as those described for delayed farletuzumab-FuG1 treatments, various control and FuG1 antibodies were added either 12 h or 14 h after spike transfection to allow effective spike cellular expression (as indicated in figures and figure legends).

### Cell extract and pseudoviral spike immunoblotting.

For cellular spike processing analysis, indicated HEK-293 or ACE stable 293 cells, VERO-E6 or Calu3 cells (70% to 80% confluent) were transfected with 1 μg (unless mentioned) of WT or spike ΔRRAR (furin mutant spike) using mirus. Various indicated control and experimental FuG1 antibodies were added on cells (at indicated concentrations) 4 h post-DNA transfection. 24 h (unless indicated) lysates were prepared and separated using 8% to 10% SDS-PAGE followed by immunoblotting either using full spike antibodies (1A9 GTX 632604, AB1N1030641). For immunoblotting analysis of pseudoviral spike processing, 1 mL respective pseudotypes (rVSV-spike*ΔG, or rVSV-ΔRRAR-spike*ΔG, etc.) either generated in the presence of IgG1 control or spike targeting IgG1 antibodies (22-IgG1, 6.30-IgG1, 12.25-IgG1, 12.19-IgG1, 6.29-IgG1) or spike targeting FuG1 antibodies (22-FuG1, 6.30- FuG1, 12.25-FuG1, 12.19-FuG1, 6.29-FuG1, etc.) as indicated in figures were added on to sucrose gradient (20% wt/vol) followed by high-speed centrifugation (25,000 g, 4 h) to break open the viral proteins. The pellet was added with 2 × SDS loading dye and incubated at 95° in PCR machine for 10 min. Samples were later subjected to SDS-PAGE immunoblotting using spike, VSV-G, VSV-M, or other indicated antibodies. VSV-matrix protein (VSV-M) served the pseudoviral loading control.

### Production of replication defective SARS-CoV-2 spike (rVSV-spike*ΔG) pseudovirions.

rVSV-spike*ΔG were generated as described previously (5, 38). Replication-restricted VSV-G pseudotyped ΔG-luciferase (VSV-G*ΔG-luciferase) rVSVw/pCAGGS-G-Kan (EH1025-PM) and BHK1 cells (EH1011) were purchased from Kerafast. In addition, SARS-CoV2 S protein pseudotyped Luc reporter virions were also purchased for ready to do experiments from BPS Biosciences pseudovirus (Cat # 79942) and (Virongy (CoV2Luc-02)). Titer of these pseudovirions were determined as per manufacture recommendations. rVSV-spike*ΔG pseudovirions were generated next to VSV-G pseudovirions in culture conditions as VSV-G induced syncytia in BHK-21 cells helped timely monitoring of spike pseudovirions recovery as described earlier (5, 38). We generated rVSV-spike*ΔG with both 293T and BHK1 cells. WT and furin mutant ΔRRAR spike (ordered as gene blocks, Life Technologies) were cloned and sequence confirmed in pCAGGS-G-amp vector, kindly provided by Dr. Michael Whitt. Briefly, 10 cm plates with 80% confluence of 293T or BHK1 cells (37°C with 5% CO_2_ overnight) were transfected with pCAGGS-spike, pCAGGS-ΔRRAR-spike (or pCAGGS-VSV-G) plasmids for the respective WT spike, furin mutant spike (or VSV-G) proteins expression, using mirus transfection (MirusBio) reagent. At 16 h posttransfection, the transfected cells were transduced with replication defective VSV-G*ΔG-luciferase (Kerafast), which encodes for luciferase. Cells were further incubated for 24 h, until the majority of cells in a side-by-side VSV-G transfected manner induced CPE. After that supernatants were collected and clarified by centrifugation at 1,000 rpm for 5 min, passed through 0.45 μm PES filter, and concentrated using 10% (wt/vol) PEG 6000 and 5M NaCl. PEGylation solution was mix and incubated at 4°C overnight on gentle rocker. The precipitated pseudovirion particles were centrifuged at 4,000 rpm for 30 min and resuspended in 1 mL of culture media. Pseudovirion particles were either used immediately or aliquoted prior to storage in −80°C to avoid multiple freeze-thaws. To transduce cells with pseudovirions, ∼2 × 10^5^ 293-ACE2 cells were seeded in a 96-well plate 20 to 24 h prior to inoculation. A single aliquot of pseudovirions was used for infection. After overnight incubation, cell lysates were generated and transferred to white wall reader plated. 5 μL d-luciferin (300 μg/mL) reagent per well was added and incubated at room temperature for indicated time. The transduction efficiency was measured by quantification of the luciferase activity using a Synergy HT 96-well microplate reader (BioTek Instruments Inc., USA). All experiments were done in triplicates and repeated at least twice or more.

### Pseudovirions generation in the presence of FuG1 antibodies.

As VSV-G induces highly effective syncytia in BHK-21, replication defective pseudovirions SARS-COV-2 pseudovirions (rVSV-spike*ΔG) were generated next to VSV-G*ΔG-pseudovirions in culture conditions as described earlier (5, 38). Briefly, 10 cm plates with 80% confluence of 293T or BHK1 cells (37°C with 5% CO_2_ overnight) were transfected with pCAGGS-spike, pCAGGS-ΔRRAR-spike (or pCAGGS-VSV-G) expression plasmids for the respective WT spike, furin mutant spike (or VSV-G) proteins using mirus transfection reagent. Various spike targeting (RBD, non-RBD-binding) antibodies were added to the cultures 6 h later at indicated concentration. At 24 h posttransfection, the transfected cells were re-treated with corresponding antibodies (FuG1 or controls) along with the inoculation of luciferase encoding replication defective VSV-G*ΔG-luciferase (Kerafast). Cells were further incubated for an additional 24 h, before the rVSV-spike*ΔG, rVSV-ΔRRAR-spike*ΔG, or (rVSV-G*ΔG) containing supernatants were harvested, followed by centrifugation and filtration to eliminate cellular debris. Pseudovirion particles were either used immediately or aliquoted prior to storage in −80°C to avoid multiple freeze-thaws.

### Spike pseudoviral transduction and host cell entry assays.

Briefly, 293-ACE2 cells were seeded at a density of 60% to 70% confluence into a 96-well cell culture plate in the DMEM media. Cells were incubated at 37°C with 5% CO_2_ overnight. The next day, media was aspirated from the cells and approximately 100 mL/well of the respective pseudotypes (rVSV-spike*ΔG) either generated in the presence of IgG1 control or spike targeting IgG1 antibodies (22-IgG1, 6.30-IgG1, 12.25-IgG1, 12.19-IgG1, 6.29-IgG1) or spike targeting FuG1 antibodies (22-FuG1, 6.30- FuG1, 12.25-FuG1, 12.19-FuG1, 6.29-FuG1, etc.) as indicated in figures were added in triplicate. After 24 h post-pseudoviral transduction, media was aspirated from the cells, followed by cell lysis. The lysates were transferred to corresponding 96-well white wall, clear bottom plates. Then, 5 μL d-luciferin (300 μg/mL) reagent was added per well and incubated at room temperature for luciferase activity signal. The transduction efficiency was measured by quantification of the luciferase activity using a Synergy HT 96-well microplate reader (BioTek Instruments Inc., USA). All experiments were done in triplicates and repeated at least twice or more. Data was plotted using GraphPad Prism software.

### Crude endosomal lysate fractionation.

A discontinuous gradient of 8%, 30%, and 42% was generated to collect the pellet at 8% to 30% interface as described earlier (32). Briefly, spike transfected cells (20 h) were washed with PBS followed by whole cell lysates generation using lysis buffer (300 mM sucrose, 10 mM Tris pH 8.0. 5 mM CaCl_2_, 3 mM imidazole, pH 7.4, 1 mM EDTA, 3 mM Mg acetate, 1 mM DTT, 0.1% Triton, ddH2O, protease and phosphatase inhibitor). After stepwise centrifuge steps (500 g, 2,000 g, 3,000 g, 4°C), post-nuclear supernatants (PNS) containing various the cell organelles in suspension were recovered. Additional centrifugation could be carried out at this step to enrich nuclei. The PNS were subsequently separated by discontinuous gradient ultracentrifugation. After recovery, the sucrose concentrations of the PNS were adjusted to 40% by adding 62% sucrose, usually with 1:1.2 V/V (percent volume per volume). PNS with 40% sucrose was loaded on the bottom of an ultracentrifuge tube. This was followed by sequential overlay with 30% sucrose and 8% sucrose. Interphases of three gradients were marked the waterproofed pen. This was followed by ultracentrifugation at 210,000 g at 4°C for 2 h. After centrifugation, we detected clear milky band of membrane particles at the interface of 8% and 30% sucrose called crude endosome (CE) fraction. The interface at 30% and 42% was diffused and significantly dense. As described in literature (32), floating late endosomes, smaller size lysosomes, early endosomes and carrier recycling vesicles are concentrated at the interface between 8% and 30% sucrose (CE), while the heavy membranes like Golgi and ER and enlarged lysosomes membranes are enriched at second interface. After recovery, proteins were measured and approximately 10 mg CE samples were loaded along with total lysates (15 mg) for immunoblotting.

### ER, trans-Golgi-network, and other membrane enrichment using commercial kits.

ER and Golgi fractions from spike transfected cells were enriched using commercial Minute enrichment kits, cat# ER-036 and cat# ER-037 respectively from Invent Biotechnologies. Briefly, 293-ACE cells were grown in 150cm^2^dishes and were divided into various groups. At ∼70 to 80 confluence cells were transfected with spike. Supernatant was removed 24 h later, and cell were collected by scraping followed by low-speed centrifugation (500 to 600 × *g* for 5 min) and cold PBS wash. Cell pellet was resuspended in supplied 550 μL buffer A (manufacturer provided in the kit) and vortexed followed by transfer of cell suspension to manufacturer supplied filter cartridge and inverted few times. After that multiple centrifugation steps were carried out as per manufacturer recommendations. After separating mitochondria, ER, lysosomes and plasma membranes, 400 μL buffer B (manufacturer provided in the kit) was added to the recovered supernatant and mixed by vortexing briefly. The tube was incubated on ice for 15 min followed by additional centrifuge at 8,000 × *g* for 5 min and supernatant with secretory vesicles of trans-Golgi was transferred to a fresh tube which was later concentrated. To enrich for cis Golgi fraction additional recommended centrifugations were carried out and pellet was suspended in 1× PBS. Lysates were mixed with protease inhibitor, SDS loading dye was added. Samples were boiled at 95° for 10 min before loading on the gel for immunoblotting. Specific enrichment of Golgi fractions was confirmed by immunoblotting against GM130 (cell signaling: 12480S), golgin-97 (cell signaling: 13192S). After Golgi fractionations, the cat# ER-037 kit also enriches remaining membrane fraction which includes lysosomes, ER, late and recycling endosomes membranes. Similar procedure was carried out using ER enrichment kit, Cat# ER-036 as per manufacturer recommendations. Selective enrichment of ER fractions was confirmed by immunoblotting against ER resident chaperone GRP78/BIP (Cell signaling: 3177S). For ER-TGN transport studies, we made use of Brefeldin A (BFA). BFA was purchased from Biolegend (420601S) and a working 5 mg/mL stock solution in ethanol was prepared. For ER to Golgi transport inhibition studies of spike, cells were treated with BFA at the time of spike transfection. Then, 24 h lysates were analyzed for immunoblotting using spike antibodies along with total lysates.

### Quantitation and statistical analysis.

Data unless indicated otherwise are presented as mean ±SEM. In general, when technical replicates were shown for *in vitro* experiments, Student’s *t* test was used for statistical analysis and the same experiment was at least repeated once with similar trend observed. When data from multiple experiments was merged into one figure, statistical significance was determined by either Wilcoxon Mann-Whitney test or unpaired T-test with Welch’s correction using Graph Pad Prism 5.0 software. For all the statistical experiments *P* values, *, *P* < 0.05; **P < 0.01; ***, *P* < 0.001 were considered statistically different or specific *P* values indicated otherwise or “ns” indicates non-significant.
